# Hierarchical Porous Graphene–Iron Carbide Hybrid Derived From Functionalized Graphene-Based Metal–Organic Gel as Efficient Electrochemical Dopamine Sensor

**DOI:** 10.3389/fchem.2020.00544

**Published:** 2020-07-30

**Authors:** Eleni C. Vermisoglou, Petr Jakubec, Ondřej Malina, Vojtěch Kupka, Andreas Schneemann, Roland A. Fischer, Radek Zbořil, Kolleboyina Jayaramulu, Michal Otyepka

**Affiliations:** ^1^Faculty of Science, Regional Centre of Advanced Technologies and Materials, Palacký University Olomouc, Olomouc, Czechia; ^2^Inorganic and Metal-Organic Chemistry, Department of Chemistry and Catalysis Research Centre, Technical University of Munich, Garching, Germany; ^3^Lehrstuhl für Anorganische Chemie I, Technische Universität Dresden, Dresden, Germany; ^4^Department of Chemistry, Indian Institute of Technology Jammu, Jammu, India

**Keywords:** graphene, dopamine, gel, metal–organic gel, nanocomposite, sensing

## Abstract

A metal–organic gel (MOG) similar in constitution to MIL-100 (Fe) but containing a lower connectivity ligand (5-aminoisophthalate) was integrated with an isophthalate functionalized graphene (IG). The IG acted as a structure-directing templating agent, which also induced conductivity of the material. The MOG@IG was pyrolyzed at 600°C to obtain MGH-600, a hybrid of Fe/Fe_3_C/FeO_x_ enveloped by graphene. MGH-600 shows a hierarchical pore structure, with micropores of 1.1 nm and a mesopore distribution between 2 and 6 nm, and Brunauer–Emmett–Teller surface area amounts to 216 m^2^/g. Furthermore, the MGH-600 composite displays magnetic properties, with bulk saturation magnetization value of 130 emu/g at room temperature. The material coated on glassy carbon electrode can distinguish between molecules with the same oxidation potential, such as dopamine in presence of ascorbic acid and revealed a satisfactory limit of detection and limit of quantification (4.39 × 10^−7^ and 1.33 × 10^−6^ M, respectively) for the neurotransmitter dopamine.

**Graphical Abstract:**
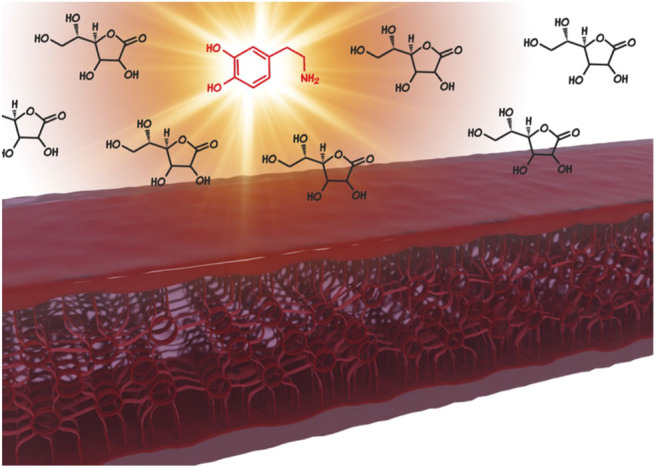
Metal-organic gel integrated with an isophthalate functionalized graphene gives rise to a hierarchically porous hybrid Fe/Fe_3_C/FeO_*x*_ enveloped by graphene, which enables the highly selective sensing of the neurotransmitter dopamine in presence of ascorbic acid in ratios similar to those of blood serum.

## Introduction

Metal–organic frameworks (MOFs) consisting of organic linkers bridging metal or metal–oxo clusters entail profound benefits when they are used in a variety of applications, such as separations, gas storage, chemical sensing, and catalysis (He et al., 2019; Chen et al., 2020; Huang et al., 2020; Roztocki et al., 2020). Nevertheless, their stability under certain environmental conditions and poor conductivity constitute limiting factors and pose a necessity for new strategies in MOF synthesis and design (Yuan et al., 2018). Toward this, gel-derived MOF synthesis remains a significantly unexplored path. This strategy meets the demand of stability by screening the inorganic metal ion and the organic struts both thermally and chemically inside the inert gel matrix. Crystallization takes place in a controlled way into the stable gel environment, and an *in situ*–formed MOF is released finally. Moreover, gel-to-crystal transformation provides a way for the construction of more multicomponent functional materials and preserves organization even in the case of incorporation of a nanomaterial, such as graphene that could improve the conductivity and modulate the architecture of the hybrid material (Aiyappa et al., 2015; Zhao et al., 2015).

Metal–organic frameworks can also constitute a sacrificial template toward the construction of metal–metal oxide or metal carbide nanoparticles via thermal treatment, for example, calcination or pyrolysis (Horike et al., 2009; Wu et al., 2014; Kornienko et al., 2015; Guan et al., 2016; Tian et al., 2017; Jayaramulu et al., 2019). Tailoring structures by tuning the microporosity and mesoporosity of the carbonaceous precursor material, for example, by integrating graphene into the metal–organic architecture, and size control of the produced nanoparticles are issues of critical importance for the properties of the final product and its specific applications (Bak et al., 2015; Zhu et al., 2017; Li et al., 2018a,b). The optimization of these two parameters is anticipated to adjust electron and mass transport, fundamental for application in biosensing (Urbanová et al., 2018; Wang et al., 2018).

The synergistic action of graphene combined with metal–metal carbide–metal oxide (M/M_3_C/MO_x_) nanoparticles provides simultaneously highly active catalytic sites and an excellent electron–transfer interface (Zhang et al., 2018). More specifically, metal-containing nanoparticles interact with the biological analytes, which can act as electron acceptors/donors, whereas the highly conductive graphene allows fast electron transfer kinetics that produces current and triggers fast response time (Gao et al., 2018; Ghanbari and Bonyadi, 2018). Moreover, chemical stability of graphene in acidic and alkaline environment, along with biocompatibility, non-toxicity, colloidal stability, and magnetic properties of metal–metal oxide nanoparticles, might open new perspectives for biomedical applications, such as magnetic resonance imaging (Thapa et al., 2019). In order to achieve such materials, the targeted pyrolysis of carbon precursors doped with metal precursors is a common method.

Biomarkers are molecules present in the human serum, and their levels in the blood can be indicative of health issues, making the development of new biosensors central aspects of biomedical materials synthesis. Two very common biomarkers of major importance in medical science are dopamine (DA) and ascorbic acid (AA). Recent biomedical advances endow AA as a part of anticancer combination therapy (McConnell and Herst, 2014) and as an antihypertensive agent (Wang et al., 2016) and relate its deficiency to muscle atrophy and deterioration in physical performance (Takisawa et al., 2019). Monitoring of AA and DA biomarkers appears highly appealing because it enables prompt prognosis, disease diagnosis, and treatment. Among various analytical methods (Bazel et al., 2018; Baenas et al., 2019; Ma et al., 2019), the electrochemical assay method provides an ideal platform for sensitive, selective, rapid, accurate, and simultaneous determination of these biomarkers, because they coexist in biological fluids, such as blood serum. A common hurdle to pass is the similar oxidation peak potentials of AA and DA at unmodified conventional electrodes, such as glassy carbon electrodes (GCEs), which results in a voltammetric overlap response (Guo et al., 2018a; Ji et al., 2018; Thirumalai et al., 2018). Besides this, the extremely low level of DA (<1 μM) compared to the concentration of AA (100–1,000 times higher than that of DA) needs to be considered (Ghanbari and Hajheidari, 2015; Edris et al., 2018). To address these issues, as well as the slow interfacial electron transfer, chemically modified electrodes (Anu Prathap et al., 2019) have been developed as sensing platforms based on conductive polymers (Xie et al., 2017; Li et al., 2019), MOFs (Fang et al., 2018), carbonaceous nanomaterials (Luo et al., 2016; Abellán-Llobregat et al., 2018), metal/metal oxide nanoparticles (Chen et al., 2018; Zhao et al., 2019a), and their composites (Grace et al., 2019; Lin et al., 2019; Zhao et al., 2019b).

Here in this work, we present the targeted synthesis of a metal–organic gel (MOG) consisting of Fe_3_Cl(H_2_O)_2_O clusters interconnected with 5-aminoisophthalic acid (NH_2_-ip) in the presence of isophthalate functionalized graphene (IG) (Vermisoglou et al., 2019). This ligand is an isomer of 2-aminoterephthalic acid (NH_2_-bdc), which is used for the preparation of NH_2_-MIL-101(Fe) (Fe_3_Cl(H_2_O)_2_O(NH_2_-bdc)_3_). It is anticipated that, by lowering the symmetry, intentional defects are created, leading to gel formation, with the IG also acting as a structure-directing agent (IG@MOG). This combination enhanced electronic conductivity and reinforced the structure. These iron–carboxylate MOGs served as precursors for the synthesis of carbon-encapsulated M/M_3_C/MO_x_ nanoparticles on graphene via pyrolysis. The IG@MOG acted as a sacrificial template leading to iron-based derivatives of Fe/Fe_3_C/FeO_x_ with conductive graphene nanosheets. It should be noted that, by changing pyrolysis temperature, the iron, carbon, oxygen, and nitrogen composition was altered. The resultant composites (after carbonization) showed significant surface area with hierarchical (micro/meso and meso/macro) pores, which is of major importance in electrochemical applications. The resultant metal-containing graphene hybrid MGH-600 (carbonized at 600°C) was investigated for DA sensing in presence of AA and revealed a satisfactory limit of detection. This significant electrochemical performance can be attributed to synergistic effects, where IG serves as a conducting matrix and provides hierarchical pores. MGH-600 exhibited remarkable stability as the electrochemical testing after ~15 months revealed.

## Experimental

### Reagents and Materials

Graphite fluoride (C: -F, 1:1.1), 5-aminoisophthalic acid (Niso) (94%), iron(III) chloride hexahydrate puriss p.a. (≥99%), L-AA, DA hydrochloride, and potassium hexacyanoferrate(III) were purchased from Sigma-Aldrich (Prague, Czech Republic). Ethanol (absolute), *N, N*-dimethylformamide (DMF), potassium chloride, and phosphate-buffered saline (PBS, pH 7.0) were purchased from Penta (Prague, Czech republic). All reagents were used as received without further purification. All stock solutions were prepared with ultrapure water (18 MΩ cm^−1^).

### Materials Synthesis

Isophthalate fluorographene (IG): see reference labeled as FG/Niso-24 h (Vermisoglou et al., 2019).

*MOG*: 1.0 g iron(III) chloride hexahydrate and 0.3 g 5-aminoisophthalic acid were dissolved separately in 11.5 mL DMF each. Then they were mixed and stirred for 30 min. The whole was transferred to a Teflon-lined autoclave reactor [Model 4744 General Purpose Acid Digestion Vessel, 45 mL; Parr Instrument Company (Illinois, United States)]. The reactor was placed inside a preheated oven (110°C) for 24 h (BINDER ED series oven drying; Sigma-Aldrich). After cooling down a red-brownish gel came out of the reactor.

*MOG@IG*: 1.0 g iron(III) chloride hexahydrate and 0.3 g 5-aminoisophthalic acid were dissolved separately in 11.5 mL DMF each. Then they were mixed, and 0.05 g IG was added under stirring for 30 min. The whole mixture was transferred to a Teflon-lined autoclave reactor (Model 4744 General Purpose Acid Digestion Vessel, 45 mL; Parr Instrument Company). The reactor was placed inside a preheated oven (110°C) for 24 h (BINDER ED series oven drying). After cooling down, a black gel was isolated.

*MOG powder*: MOG was dispersed in 80 mL ethanol and stirred for 1.5 h. Then it was centrifuged at a speed 10,000 for 10 min (Sigma 6–16 K Centrifuge), and the supernatant was removed. This washing procedure was repeated twice, and finally, the red-brownish solid was dried in an oven at 60°C for 48 h (BINDER ED series oven drying). The dried solid was ground in an agate mortar (Aldrich).

*MOG@IG powder*: MOG@IG was dispersed in 80 mL ethanol and stirred for 1.5 h. Then it was centrifuged at a speed 10,000 for 10 min (Sigma 6–16 K Centrifuge), and the supernatant was removed. This washing procedure was repeated twice, and finally the black solid was dried in an oven at 60°C for 48 h (BINDER ED series oven drying). The dried solid was ground in an agate mortar (Aldrich).

*MGH-600*: A crucible containing ~0.1 g MOG@IG powder was placed in the center of a laboratory tube furnace equipped with a quartz glass tube (furnace type: LT 50/300/13) and heated (temperature ramp 5°C/min) under N_2_ flow (50 mL/min) at 600°C for 6 h. After cooling down to room temperature, a black powder was isolated.

*MGH-400*: A crucible containing ~0.1 g MOG@IG powder was placed in the center of a laboratory tube furnace equipped with a quartz glass tube (furnace type: LT 50/300/13) and heated (temperature increase 5°C/min) under N_2_ flow (50 mL/min) at 400°C for 6 h. After cooling down to room temperature, a black powder came out.

*MGH-800*: A crucible containing ~0.1 g MOG@IG powder was placed in the center of a laboratory tube furnace equipped with a quartz glass tube (furnace type: LT 50/300/13) and heated (temperature increase 5°C/min) under N_2_ flow (50 mL/min) at 800°C for 6 h. After cooling down to room temperature, a black powder came out.

### Materials Characterization

X-ray diffraction patterns were recorded with a X'Pert PRO MPD diffractometer Malvern Panalytical (Worcestershire, United Kingdom) in the Bragg–Brentano geometry equipped with Co-Kα radiation source (40 kV, 30 mA, λ = 0.1789 nm).

Fourier transform infrared (FTIR) spectra were recorded on an iS5 FTIR spectrometer Thermo Fisher Scientific (Brno, Czech Republic) using the Smart Orbit ZnSe ATR accessory. Briefly, a droplet of an ethanolic dispersion of the test powder material was placed on a ZnSe crystal and left to dry and form a film. Spectra were acquired by summing 100 scans, using N_2_ gas flow through the ATR accessory.

X-ray photoelectron spectroscopy (XPS) was carried out with a PHI VersaProbe II [Physical Electronics (Münich, Germany)] spectrometer using an Al Kα source (15 kV, 50 W). The obtained data were evaluated with the MultiPak (Ulvac; PHI, Inc.) software package.

Raman spectra were collected using a DXR Raman spectroscope (Thermo Scientific [Brno, Czech Republic)] equipped with a laser operating at a wavelength of 633 nm.

The ^57^Fe zero-field Mössbauer spectra were recorded at room temperature employing a Mössbauer spectrometer (MS2007) operating in a constant acceleration mode and equipped with a 50 mCi ^57^Co(Rh) source (Pechousek et al., 2010; Pechoušek et al., 2012). The acquired ^57^Fe Mössbauer spectra were processed (i.e., noise filtering and fitting) using the MossWinn software program (Klencsár et al., 1996). The values of the isomer shift were referred to α-Fe foil sample at room temperature.

### Magnetic Measurements

Samples were analyzed using a Quantum Design Physical Properties Measurement System [PPMS DynaCool system (Darmstadt, Germany)] with the vibrating sample magnetometer option. The experimental data were corrected for the diamagnetism and signal of the sample holder. The temperature dependence of the magnetization was recorded in a sweep mode of 1 K/min in the zero-field-cooled (ZFC) and field-cooled (FC) measuring regimens. To get the ZFC magnetization, the sample is first demagnetized at a temperature higher than the blocking temperature (magnetic moments of all particles are randomly oriented), and after that, it is cooled down without applied magnetic field to a temperature lower than the blocking temperature. In the last step, an external magnetic field (1,000 Oe) is applied, and the magnetization is recorded upon warming the sample. To obtain the FC magnetization curve, almost the same process is performed, but with applied external magnetic field during cooling of the sample.

Surface area and pore size analyses were performed by means of N_2_ adsorption/desorption measurements at −196°C, on a volumetric gas adsorption analyzer [3Flex; Micromeritics (Norcross, Georgia, United States)] up to 0.9626 bar. The sample was degassed under high vacuum (7 × 10^−2^ mbar) for 12 h at 110°C, whereas high-purity (99.999%) N_2_ and He gases were used. The Brunauer–Emmett–Teller (BET) area was determined assuming a molecular cross-sectional area of 16.2 Å^2^ for N_2_ (−196°C). The isotherms were further analyzed by means of non-local Density Functional Theory (DFT) slit pore kernels for N_2_.

The samples were also analyzed by scanning electron microscopy (SEM) on a Hitachi SU6600 instrument (Mannheim, Germany) with an accelerating voltage of 5 kV. Transmission electron microscopy (TEM) images were obtained on a JEOL 2100 (Tokyo, Japan) instrument equipped with an LaB6-type emission gun operating at 200 kV. STEM-HAADF (high-angle annular dark-field imaging) analyses for elemental mapping of the products were performed with an FEI Titan HRTEM (Tokyo, Japan) (high-resolution TEM) microscope operating at 200 kV. For these analyses, a droplet of a dispersion of the material in ethanol with concentration of ~0.1 mg mL^−1^ was deposited onto a carbon-coated copper grid and dried.

### Sensing Measurements

All electrochemical measurements were performed using a PGSTAT128N potentiostat (Metrohm Autolab B.V., Prague, Czech Republic) monitored by NOVA software (version: 1.11.2). A typical three-electrode configuration was used. Bare or MGH-600 sample modified GCEs were used as working electrodes; a saturated Ag/AgCl (2Theta, Český Tešín, Czech Republic) and a platinum wire electrode served as reference and counter-electrode, respectively. Electrochemical impedance spectroscopy (EIS) measurements were performed in 0.1 mol L^−1^ KCl electrolyte containing 5 mmol L^−1^ K_3_Fe(CN)_6_ redox probe; otherwise, PBS (pH 7.0) was used as a supporting electrolyte. Glassy carbon electrodes were modified as follows. They were first polished on wet silicon carbide paper using 1 and 0.05 μm Al_2_O_3_ powder sequentially and then washed in water and ethanol for a few minutes, respectively. Thereafter, 10 μL drop of MGH-600 aqueous suspension in (2 g L^−1^) was coated onto the GCE surface and allowed to dry at laboratory temperature to form a thin film.

## Results and Discussion

**Scheme 1:**
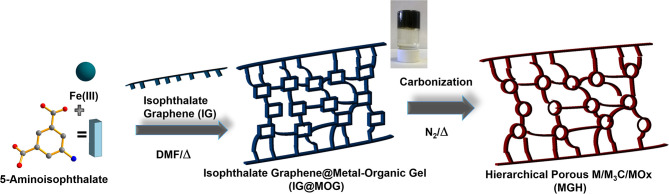
Schematic illustration of IG-based metal–organic gel (MOG@IG) (inset shows optical image of black gel), which produced (after solvent removal) hierarchical porous metal–metal carbide–metal oxide composite (M/M_3_C/MO_x_) under carbonization at various temperatures.

The solvothermallly prepared iron organic gel without graphene had a dark red-brownish color (Scheme S1), whereas in the presence of isophthalate graphene (IG), a black gel was formed (Scheme 1). The DMF present in the Teflon-lined autoclave was completely immobilized in the porous gel, as verified by the “tube inversion” method (Scheme S2). Isophthalate graphene was used in order to direct *in situ* the interfacial gelation along the surface of IG through coordination of the metal clusters with the isophthalate groups anchored to the graphene. Moreover, it acted as a physical cross-linker and as a directing agent for the formation of the porous structure. Isophthalate graphene served as a structural reinforcement nanofiller, triggering the growth of robust ordered lamellar structures and increased conductivity. The MOG@IG displayed “honeycomb” porous three-dimensional (3D) architecture (Scheme 1) (Wang et al., 2015; Vermisoglou et al., 2018). Isophthalate graphene's role as a structure-directing agent could be fulfilled up to a certain concentration, while excessive amounts (e.g., double the amount used in this work) inhibited the gelation process. A 3D MOG network is anticipated to be formed by a self-assembly process in between IG interfaces where the metal would be coordinated to the organic linkers, and other non-covalent interactions occur between the constituents, such as hydrogen bonding or π-π stacking, which attributes to the profound gelation ability (Jayaramulu et al., 2017). Metal–organic frameworks are built up by various metals and organic linkers to produce 3D crystalline porous coordination networks under coordination continuation conditions. However, under coordination perturbation conditions, solvothermal treatment will lead to MOG formation (Li et al., 2013). The coordination of 5-aminoisopthalate and iron ion clusters yields octahedrally coordinated Fe^3+^ with carboxylate oxygen atoms because of the stronger coordination of Fe-O compared to Fe^3+^ cation- aqueous solvent interactions. Therefore, Fe^3+^ metal ions and 5-aminoisopthalate form MOF-like structure, which aggregates to an amorphous MOG (Scheme S1). The introduction of IG disrupts the MOF crystal growth, enhancing mismatch growth over oriented crystallization, where the MOF nanoparticles initially undergo controlled growth and are selectively chelated with oxygen functionalities of IG sheets (Scheme 1).

**Figure 1:**
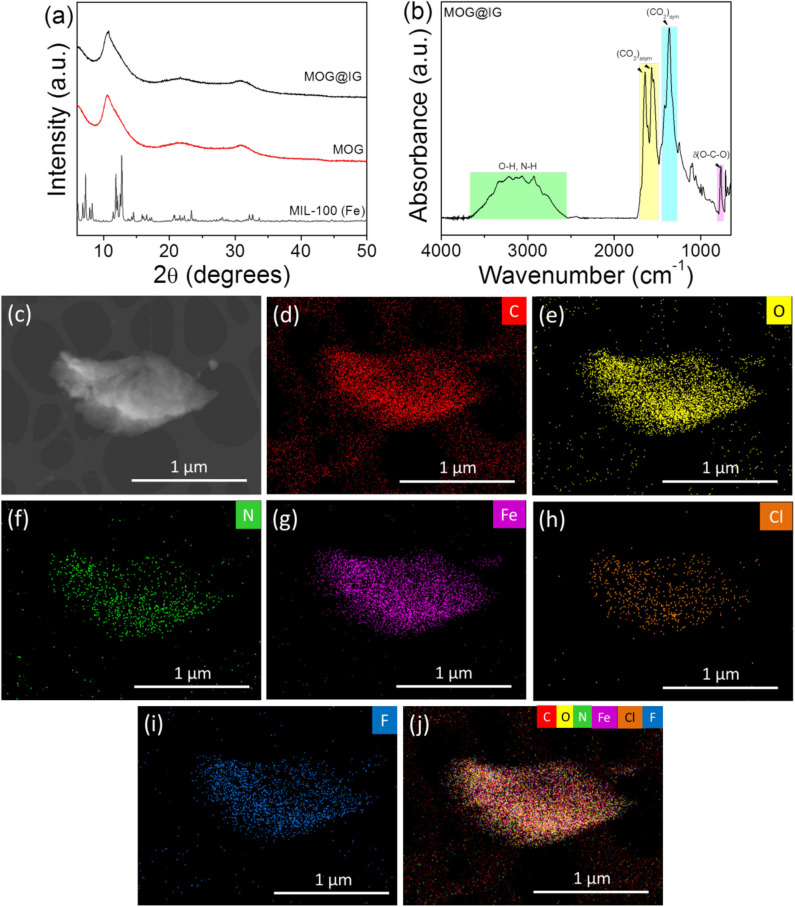
**(a)** Simulated XRD pattern of MIL-100(Fe) and experimental XRD patterns of MOG and MOG@IG powders; **(b)** FTIR spectrum of MOG@IG powder; **(c)** dark-field HRTEM image of an MOG@IG flake used for EDS chemical mapping: **(d)** carbon map, **(e)** oxygen map, **(f)** nitrogen map, **(g)** iron map, **(h)** chlorine map (Cl acts as counter-anion stemming from FeCl_3_·6H_2_O precursor), **(i)** fluorine map (F stemming from IG), and **(j)** overall map of all these elements.

By removing the DMF solvent from the MOG (Scheme S1) by a mild and slow-drying process, the as-produced MOG powder was obtained. The powder XRD shows broad reflections, and it clarifies the amorphous behavior (Figure 1a). The participation of carboxylate groups in bonds with Fe^3+^ was verified by FTIR spectroscopy data. In MOG@IG (powder) spectrum (Figure 1b), the observed vibrational frequencies ν_asym_ (COO^−^) (1,646 and 1,569 cm^−1^) and ν_sym_ (1,369 cm^−1^) for the carboxylate ligands indicate on the coordination of the carboxylic acid groups with the metal ions through deprotonation (Vermisoglou et al., 2014). Fourier transform infrared spectra of MOG, as well as the parent materials, are presented in Figures S1A–C. X-ray photoelectron spectroscopy survey data, high-resolution C 1s, Fe 2p, and O 1s spectra are presented in Figures S2A–D, whereas the atomic percentages (at. %) of various functional groups present in IG and MOG@IG composite obtained from deconvolution of the high-resolution C 1s XPS spectrum are summarized in Table S1. The C1s XPS spectrum of MOG@IG powder compared to that of IG (Figure S2B) clearly indicates the presence of the ligand functional groups. Moreover, from high-resolution Fe 2p XPS spectrum (Figure S2C), the fingerprint of Fe^3+^ electronic structures and the absence of Fe^2+^ were verified, whereas the peak separation ~13.9 eV was equal to the peak separation of MIL-100(Fe) attributed to α-Fe_2_O_3_ nodes in the MOG framework. Further, in the high-resolution O 1s XPS spectrum (Figure S2D) the peaks at 530.1, 531.8, and 533.3 eV are assigned to the Fe-O, O=C, and O–C bonds in MOG@IG.

The Raman spectrum of MOG powder (Figure S3, bottom) has the typical characteristic signals of MIL-100 (Fe), as reported in the literature (Lohe et al., 2009; Wan et al., 2018; Gong et al., 2019). These findings along with FTIR data support MIL-100 (Fe)–like features in the MOG material. In the presence of graphene (Figure S3, top), the graphene characteristics prevail in the Raman spectrum of MOG@IG as it is documented by the D and G peaks. The intensity ratio of D to G signal exceeds 1 (i.e., 1.16) anticipated for covalently functionalized graphene (Vermisoglou et al., 2019). In order to confirm the purity of MOG@IG sample, ^57^Fe Mössbauer spectroscopy was used (Figure S4), and Mössbauer hyperfine parameters, derived from spectra fitting, are summarized in Table S2. The spectral profile can be decomposed to the one doublet component corresponding to the Fe^3+^ ions octahedrally coordinated in a high-spin state (*S* = 5/2).

Figure S5A depicts a typical SEM image of MOG@IG before solvent removal, where a sponge-like morphology is observed. In the corresponding TEM image of MOG@IG (Figure S5B), IG flakes can be observed in the gel environment and are also present in MOG@IG powder after the solvent removal (Figure S5C). Dark-field HRTEM images of an MOG@IG flake were used for energy-dispersive X-ray spectroscopy (EDS) elemental mapping (Figure 1c). Energy-dispersive X-ray spectroscopy density maps (Figures 1d–j) revealed a dense and homogeneous distribution of all the involved elements, that is, C, O, N, Fe indicating uniformly coexisting MOG and IG. This implies highly organized structures where the accommodation of MOG structure between the graphene layers or alternatively the incorporation of graphene into MOG is homogeneous.

**Figure 2:**
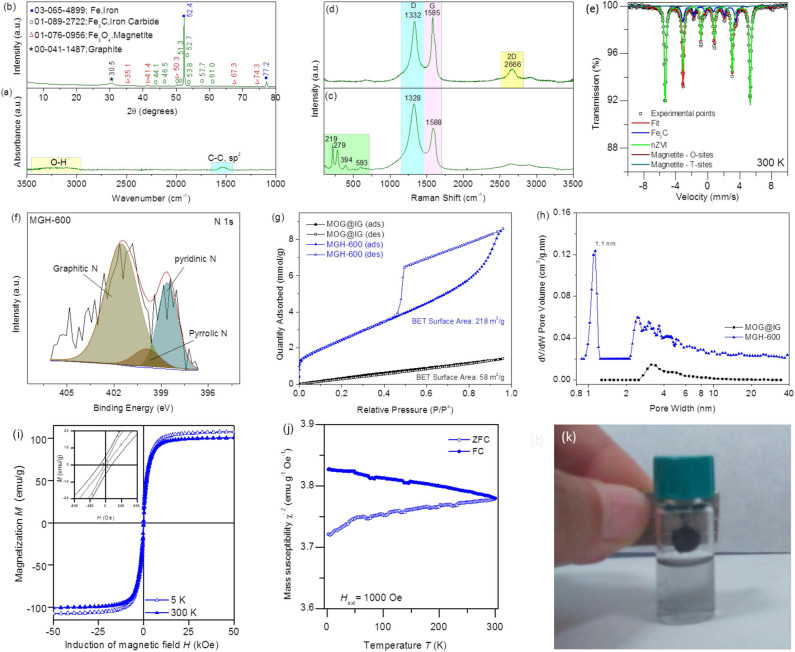
**(a)** FTIR spectrum and **(b)** XRD pattern of MGH-600; **(c)** Raman spectrum on a MGH-600 particle (bottom) and **(d)** on graphene flake (top) of MGH-600; **(e)** Mössbauer spectrum of MGH-600; **(f)** High-resolution N 1s spectrum of MGH-600; **(g,h)** N_2_ sorption isotherms of MOG@IG powder and MGH-600 and the corresponding PSD; **(i,j)** Magnetization and mass susceptibility measurements of the MGH-600 sample; **(k)** MGH-600 (dispersed in ethanol) behavior under the application of a magnetic field.

MOG@IG powder was subjected to carbonization at 600°C for 6 h under N_2_ atmosphere in order to improve properties, such as BET surface area and conductivity, which would upgrade its performance, for example, in electrochemical sensing applications. The FTIR spectrum of the as-produced MGH-600 (Figure 2a) reveals C sp^2^ hybridization and absence of the characteristic functional MOG groups. The XRD pattern of MGH-600 XRD (Figure 2b) confirms the presence of graphene (low intense broad reflection at 2θ = 30.5°, Co k-alpha radiation λ = 1.9373 Å) of Fe, iron carbide (Fe_3_C), and magnetite (Fe_3_O_4_) (JCPDS cards no. 00-041-1487 for graphite, No. 03-065-4899 for Fe, no. 01-089-2722 for Fe_3_C, and no. 00-076-0956 for Fe_3_O_4_). Fourier transform infrared spectra and XRD patterns of the samples MGH-400 and MGH-800, where the carbonization temperatures were 400 and 800°C, are presented in Figures S1E–G, S6.

Raman spectroscopy can provide further insight on the structure of the graphite-encapsulated nanoparticles (Figure 2c) and on the graphene flakes (Figure 2d) present in the composite material MGH-600. Both spectra bear the characteristic graphene D (~1,332 cm^−1^) and G (~1,585 cm^−1^) bands assigned to disorder/defects and C sp^2^, but the spectrum of the encapsulated nanoparticles exhibits in the range 200–600 cm^−1^ the iron carbide and/or zero valent iron peaks, with the latter possibly being overlapped by the former (Liu et al., 2014; Alahmadi and Siaj, 2018). Moreover, the spectrum of the graphene flake reveals a symmetrically shaped 2D peak (~2,666 cm^−1^) implying few layered graphene flakes. This is also supported by an intensity ratio *I*_D_/*I*_G_ ~0.9 < 1. Further increase of the carbonization temperature to 800°C triggers an even more intense symmetric and low full width at half maximum 2D band, indicating fewer graphene layers, but this improvement in graphene quality is accompanied by a larger size of iron-containing nanoparticles (see Figures S7–S9; Raman spectrum, SEM images, and TEM images, respectively). To get a deeper insight into the chemical nature of the nanoparticles, ^57^Fe Mössbauer spectroscopy at room temperature was employed (Figure 2e). A fitting model consisting of four sextets was adopted to correctly describe the spectrum profile. The dominant sextet component reflects the presence of zero valent iron nanoparticles (nZVI) in the system with determined Mössbauer hyperfine parameters depicted in Table S2. The sextet with lowest value of hyperfine magnetic field belongs to the Fe_3_C nanoparticles present in the system. The last two sextets in the spectrum confirm the presence of magnetite, where the sextet with the higher magnetic hyperfine field defining the Fe^3+^ ions located in the tetrahedral sites, whereas the sextet with the lower values of the hyperfine magnetic field is attributed to the Fe^2+^ and Fe^3+^ ions occupying the octahedral sites in the magnetite spinel crystal structure. Mössbauer spectra of MGH-400 and MGH-800 are illustrated in Figure S10.

The XPS survey spectra reveal a nitrogen content of ~4.1 at. %, and the high-resolution XPS N1s spectrum (Figure 2f) contains three peaks at 398.6, 399.9, and 401.5 eV corresponding to pyridinic N, pyrrolic N, and graphitic N, implying nitrogen-doped MGH-600 in accordance with the literature (Guo et al., 2018b). The BET-specific surface areas derived from nitrogen adsorption/desorption isotherms were 58 and 218 m^2^/g for MOG@IG powder and MGH-600, respectively (Figure 2g). Mesopores still exist in MGH-600, but also extended microporosity appears with pore size distribution centered at ~1.1 nm (Figure 2h). During the carbonization process, the 5-aminoisophthalate linkers present in the MOG@IG act not only as N source leading to disrupted N-doped graphene but also an etching source due to ammonia gas that is produced from polycondensation reactions creating thus microporosity (Tang et al., 2019). Nitrogen sorption isotherms, BET surface areas, and the corresponding Pore Size Distribution (PSD) for all samples are presented in Figures S11, S12.

To unveil the magnetic properties of the MGH-600 sample, hysteresis loops and ZFC/FC magnetization curves were recorded (Figures 2i,j). The magnetization vs. applied field at room temperature does not show a hysteresis (Figure 2i) indicating superparamagnetic behavior of the nanoparticles, where the spins of all magnetic nanoparticles fluctuate between the orientations of the easy axis of magnetization. As the temperature decreased to 5 K, the sample shows certain values of coercivity (*H*_C_) and remanence (*M*_R_) reflecting that the system is in a blocking state below transition temperature (Tuček et al., 2006). The saturation magnetization of nZVI (~130 emu/g) is reduced with respect to the value commonly observed for nZVI (~200 emu/g) due to normalization to the weight of the sample also containing Fe_3_C nanoparticles and a carbon scaffold. Nevertheless, the system shows a very strong magnetic response as evidenced by reaching the magnetic saturation under small applied magnetic fields (below 1 T, see Figure 2i).

From the magnetic point of view, the blocked state is very often also documented by a maximum at the ZFC magnetization curve recorded during the ZFC-FC measurement (Figure 2j). The onset of a maximum in the ZFC curve is significant around 50 K, suggesting the presence of a superparamagnetic fraction. The superparamagnetic fraction is probably composed of Fe_3_C nanoparticles, because the rest of the ZFC-FC curves are dominated by the usually observed response of nZVI nanoparticles. MGH-600's behavior under the application of a magnetic field is illustrated in Figure 2k.

**Figure 3:**
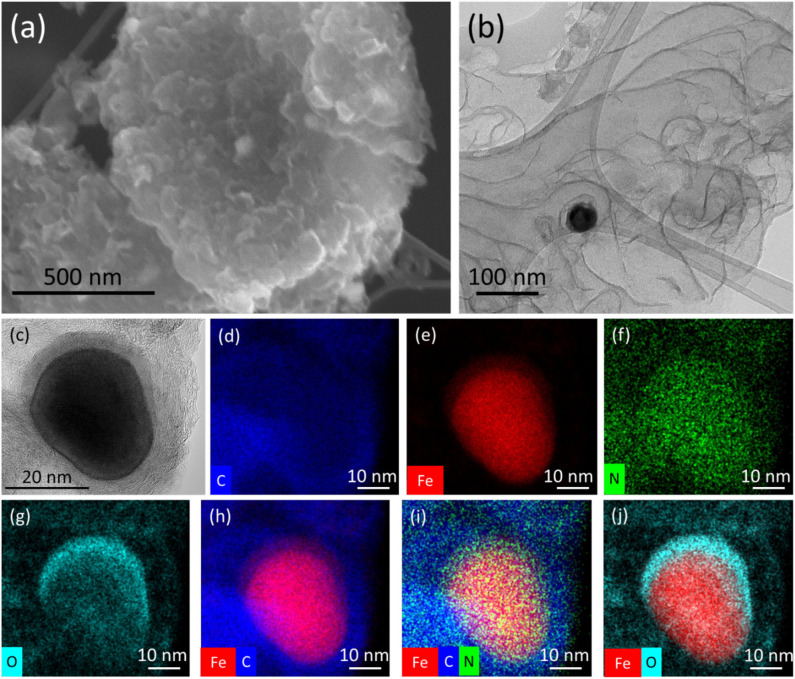
**(a)** SEM and **(b)** TEM morphological characteristics of MGH-600; **(c)** HRTEM image of an iron-containing nanoparticle of MGH-600 and the corresponding EDS chemical mapping: **(d)** carbon map, **(e)** iron map, **(f)** nitrogen map **(g)** oxygen map, and overall maps of **(h)** iron and carbon, **(i)** iron, carbon and nitrogen, and **(j)** iron and oxygen elements.

Figure 3a shows an SEM image of the MGH-600 material, which comprised curved sheets, and because of this morphology, restacking is prevented, resulting thus in a high BET-specific surface area. Furthermore, the presence of carbon-encapsulated iron/iron carbide/iron oxide nanoparticles on the graphene interfaces hinders aggregation and restacking. Such a nanoparticle can be observed in the TEM micrographs (Figure 3b) with a size close to 50 nm. In the same image, thin almost-transparent folded graphene sheets are observed, which are indicative of an extensively exfoliated material. This extended exfoliation could be attributed (a) to the initially functionalized graphene that was used for the synthesis of MGH-600; (b) to solvothermal conditions, where under high temperature and pressure the solvent molecules penetrate between the graphene sheets; and mostly (c) to pyrolysis conditions, where decarboxylation and condensation reactions take place, and gaseous species, such as NH_3_ or CO_2_ can be rapidly developed in parallel with iron-containing nanoparticles forming. Going from MOG@IG to carbon-encapsulated nanoparticles has an additional advantage that both the metal and the carbon source are present in the parent material. A TEM image of an iron-containing particle is presented in Figure 3c, and EDS density maps (Figures 3d–i) revealed the presence of carbon and nitrogen extending throughout the image also out of the particle area, whereas oxygen is more dense on the upper part of the particle as if the iron-containing particle is surrounded by an N-doped graphitic layer and preferentially oxide on the top (Figure 3j). The nitrogen mapping is an additional evidence of the presence of N-doped graphene.

**Figure 4:**
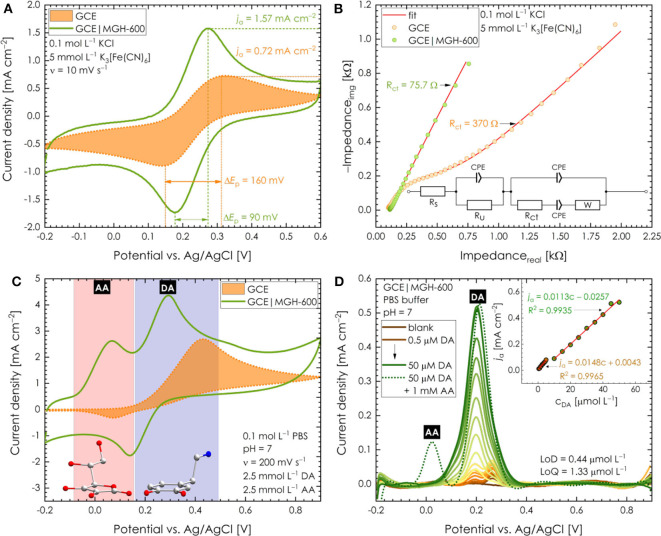
**(A)** CV response of bare GC electrode and GC electrode modified with MGH-600 sample in the presence of 0.1 mol L^−1^ KCl electrolyte containing 5 mmol L^−1^ ferricyanide(III). **(B)** EIS response of bare GC electrode and GC electrode modified with MGH-600 sample in the presence of 0.1 mol L^−1^ KCl electrolyte containing 5 mmol L^−1^ ferricyanide(III); inset: modified Frumkin–Melik–Gaykazyan circuit used for fitting of impedance data; additional parameters: 5-mV AC amplitude, a bias potential of 220 mV, the frequency range from 0.1 Hz to 100 kHz. **(C)** CVs response of the bare GC electrode and GC electrode modified with MGH-600 sample in the presence of PBS buffer (pH 7.0) containing both DA (2.5 mmol L^−1^) and AA (2.5 mmol L^−1^). **(D)** SWV records (after the background subtraction) of the GC electrode modified with MGH-600 sample in the presence of various concentrations of DA and constant concentration of AA (1 mmol L^−1^); additional parameters: amplitude 25 mV, step height 5 mV, frequency 25 Hz.

The electrochemical performance of MGH-600 was studied by means of cyclic voltammetry (CV), EIS, and square-wave voltammetry (SWV). This material was chosen to be studied thoroughly among MGH-400 and MGH-800 because the CV response of the GCE modified with different MGH samples in the presence of PBS buffer (pH 7.0) containing both DA (*c*_DA_ = 2.5 mmol L^−1^) and AA (*c*_AA_ = 2.5 mmol L^−1^) had the maximum ability to facilitate the electron transfer and allow the separation of both molecules (Figure S13). This could be attributed to a “balance” between graphene formation and particle size, giving thus rise to a high BET surface area (Figure S11). As the pyrolysis temperature increased from 400 to 800°C, the qualitative Raman spectra characteristics of graphenes, such as low *I*_D_/*I*_G_ intensity ratio and a symmetric intense 2D peak were improved (Figure 2d and Figure S7). Nevertheless, this improvement was accompanied by iron-containing nanoparticle aggregation as it can be observed in TEM images (Figure S9). The threshold for having graphene nature quality avoiding nanoparticle aggregation corresponded to pyrolysis temperature of 600°C. This can be also supported by the BET surface area that was maximized at this temperature (Table S3), as well as the performance in CV measurements (Figure S13). Figure 4A shows a set of CVs recorded with the bare GCE and GCE modified with MGH-600 sample in the presence of 0.1 mol L^−1^ KCl electrolyte containing 5 mmol L^−1^ ferricyanide(III) as an electroactive probe. As can be seen, the bare GCE exhibits a faradaic response of ferricyanide(III) giving the current density of *j*_a_ = 0.72 mA cm^−2^ (peak-to-peak separation Δ*E*_p_ = 160 mV). As it is evident, the presence of the MGH-600 sample can significantly boost the voltammetric performance (*j*_a_ = 1.57 mA cm^−2^) and reduce the value of peak-to-peak separation up to Δ*E*_p_ = 90 mV to make the electrochemical process more reversible. It is very well-known that the estimation of the heterogeneous electron transfer rate constant, *k*^0^, has a paramount importance when the electrochemical performance of a novel material is examined (Randviir, 2018). Because of this reason, a method of EIS was employed. A closer inspection revealed that the GCE modified with MGH-600 exhibits significantly lower value of charge-transfer resistance (*R*_ct_ = 75.7 Ω) compared to the unmodified GCE (*R*_ct_ = 370 Ω). It should be noted that the *R*_ct_ value calculated from the equivalent circuit fitting in EIS (for circuit details, see inset in Figure 4B) is inversely proportional to the exchange current density via the equation (Randviir and Banks, 2013; Randviir, 2018):

$$\begin{array}{l} R_{ct}=\frac{RT}{nFi_{0}}, \end{array}$$

where *i*_0_ = *nFAk*^0^*c*. Following this fact, one can conclude that

$$\begin{array}{l} R_{ct}=\frac{RT}{n^{2}F^{2}Ack^{0}}. \end{array}$$

Considering the aforementioned *k*^0^ was estimated to be 2.03 × 10^−3^ and 9.95 × 10^−3^ cm s^−1^ for bare GC and GCE modified with the MGH-600 sample, respectively. As it stands, the obtained EIS results are in good agreement with those obtained by CV and prove that MGH-600 has the ability to enhance electron transfer. Such an improvement can be explained by the structure of MGH-600. As mentioned previously, MGH-600 exhibits a highly porous structure where carbon atoms in *sp*^2^ hybridization prevail. Such findings together with the fact that the material itself is *N*-doped create a plausible explanation of the nature of this highly conductive system. Furthermore, the MGH-600 sample was tested as a promising catalyst for the electrochemical determination of DA and AA (further abbreviated as DA and AA, respectively). Figure 4C displays a CV response of the bare GCE and GCE modified with MGH-600 in the presence of PBS buffer (pH 7.0) containing both DA (2.5 mmol L^−1^) and AA (2.5 mmol L^−1^). It is possible to recognize that with the bare GCE there is no chance to separate both molecules, as discussed in the *Introduction*. On the contrary, MGH-600 has the great ability to facilitate the electron transfer and then allows the separation of both analytes. While SWV offers higher sensitivity and good separation background current, we decided to use this technique to further study the DA detection. Figure 4D shows the typical SWV record (after background subtraction) of the GCE modified with MGH-600 in the presence of various concentrations of DA. As can be seen, the current density showed a good linear relationship vs. the DA concentration and was expressed as *j*_a_ = 0.0113c – 0.0257 (*R*^2^ = 0.9935) and *j*_a_ = 0.0148c – 0.0043 (*R*^2^ = 0.9965) in the ranges of 0.5–5 and 10–50 μmol L^−1^, respectively. Moreover, the addition of AA as a common interferent has no significant influence during the determination of DA. Using the first concentration range, the values for LoD (limit of detection) and LoQ (limit of quantification) were calculated to be 0.44 and 1.33 μmol L^−1^, respectively. The presence of two linear regression equations reflects the different adsorption behavior of DA at different concentration levels. From the beginning, rapid adsorption of DA on the GCE modified with MGH-600 leads to a rapid increase of the current density. On the other hand, another increase of DA concentration results in slower increase of current density. Such observation can be explained by impurities coming from the oxidation processes of DA, which reduce the kinetics of electron transfer. Further, our results are comparable with various MOF-derived/iron-based hybrid nanocomposite materials in the literature in terms of performance in DA sensing (Table S4). To show the practical applicability of the MGH-600 sample as a successful sensing platform, a stability test was performed using CV. Figure S14 depicts that even after 15 months the electrode modified with the MGH-600 sample exhibits similar current response toward DA determination as freshly synthesized sample (the drop of current density was found to be 13.1% only). Moreover, the 15-months-old sample still has the same ability as the fresh sample to distinguish between both DA and AA molecules. This sample was also investigated microscopically after this term. The TEM images (Figure S15) illustrate graphene sheets almost transparent with few aggregates of iron-containing nanoparticles. This justifies the small drop of performance in electrochemical testing and confirms the significant stability of sample.

## Conclusions

In summary, synthesis of gel-derived MOG network with homogeneously incorporated isophthalate graphene participating as a structure-directing agent led in porous honeycomb lamellar structures. The graphene incorporation in a uniform way into this structure enriches its properties with all the outstanding properties of graphene especially regarding conductivity and specific surface area. This material acts as a scaffold and by carbonization results in magnetic graphene hybrids that could have a wide range of potential applications, among them highly selective sensing of DA in presence of AA in ratios similar to those of blood serum. The stability of these hybrids based on the inertness of graphene sheets and the carbon-encapsulated M/M_3_C/MO_x_-containing particles render them exceptionally appealing both for biosensing and magnetic applications.

## Data Availability Statement

The datasets presented in this study can be found in online repositories. The names of the repository/repositories and accession number(s) can be found below: https://data.mendeley.com/datasets/958n75m59g/draft?a=5f81a7c5-09b4-401d-a9f6-1b1fb56912fe.

## Author Contributions

EV, PJ, RZ, RF, KJ, and MO designed the study. EV and KJ synthesized the materials. EV, OM, VK, and AS characterized the materials. EV interpreted FTIR and Raman spectra and interpreted FTIR, XPS, Raman, BET, microscopy and mapping, and XRD. OM performed and interpreted the magnetic and Mössbauer measurements. VK performed and interpreted BET measurement. AS provided the mechanistic insights. PJ carried out and interpreted the electrochemical experiments. All authors contributed to the article and approved the submitted version.

## Conflict of Interest

The authors declare that the research was conducted in the absence of any commercial or financial relationships that could be construed as a potential conflict of interest.
