# Supplementary material for: Hierarchical Porous Graphene–Iron Carbide Hybrid Derived From Functionalized Graphene-Based Metal–Organic Gel as Efficient Electrochemical Dopamine Sensor
Source: Front Chem. 2020 Jul 30;8:544. doi: 10.3389/fchem.2020.00544 (PMC7409389; doi:10.3389/fchem.2020.00544)
Supplement: Supplementary file 1 [file Data_Sheet_1.docx]

**Supporting Information**

**to**

**Hierarchical Porous Graphene-Iron Carbide Hybrid Derived from Functionalized Graphene-Based Metal-Organic Gel as Efficient Electrochemical Dopamine Sensor**

Eleni C. Vermisoglou^1†^, Petr Jakubec^1†^, Ondřej Malina^1^, Vojtěch Kupka^1^, Andreas Schneemann^2,3^, Roland A. Fischer^2^, Radek Zbořil^1^, Kolleboyina Jayaramulu^1,2,4^ and Michal Otyepka^1*^

^1^ Faculty of Science, Regional Centre of Advanced Technologies and Materials, Palacký University Olomouc, Olomouc, Czechia, ^2^ Inorganic and Metal-Organic Chemistry, Department of Chemistry and Catalysis Research Centre, Technical University of Munich, Garching, Germany, ^3^ Lehrstuhl für Anorganische Chemie I, Technische Universität Dresden, Bergstr, Germany, ^4^ Department of Chemistry, Indian Institute of Technology Jammu, Jammu & Kashmir, India

Corresponding author: [michal.otyepka@upol.cz](mailto:michal.otyepka@upol.cz)

^†^ Equally contributed this work.


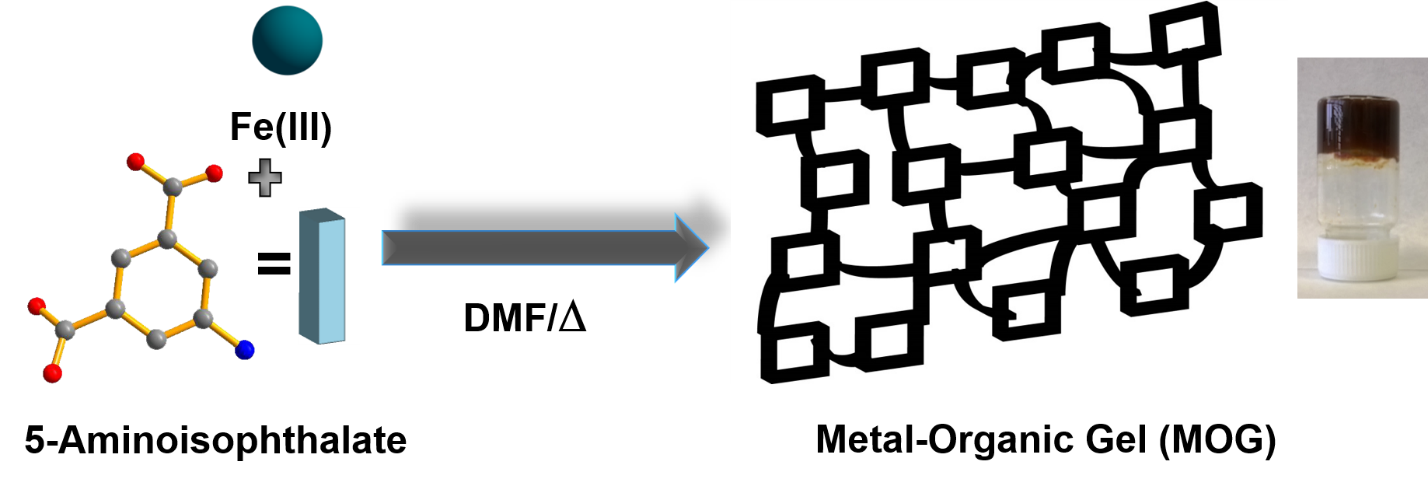


**SCHEME S1.** Schematic Illustration of synthesis metal-organic gel by using iron (III) and 5-aminoisopthalate.

**
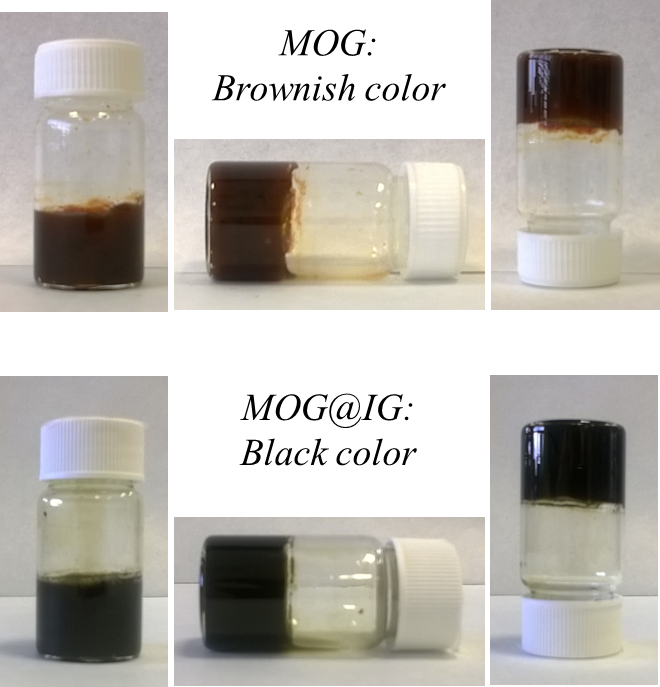
**

**Scheme S2.** Tube inversion method: A gel prepared in a vial/tube was inversed in order to see if there is a flow of the solvent or if the solvent is immobilized (gel). This is a very common test that is used widely in literature related to gel preparation (Huynh et al., 2017, Nguyen et al., 2014).

Huynh, C. T., Zheng, Z., Nguyen, M. K., McMillan, A., Tonga, G. Y., Rotello, V. M., et al. (2017). Cytocompatible Catalyst-Free Photodegradable Hydrogels for Light- Mediated RNA Release To Induce hMSC Osteogenesis. *ACS Biomater. Sci. Eng.* 3, 2011-2023. <https://pubs.acs.org/doi/10.1021/acsbiomaterials.6b00796>

Nguyen M. K., Jeon, O., Krebs, M. D., Schapira, D., Alsberg E. (2014). Sustained localized presentation of RNA interfering molecules from in situ forming hydrogels to guide stem cell osteogenic differentiation. *Biomaterials* 35, 6278-6286. <http://dx.doi.org/10.1016/j.biomaterials.2014.04.048>

**FTIR spectroscopy**

In **Figures S1c, S1d** the absence of the characteristic band at 1680-1730 cm^-1^, related to the protonated carboxylate group, indicates complete deprotonation of the ligand Niso in MOG, MOG@IG powder samples and implies metal coordinating carboxylate groups (Zhang et al., 2007). The absorption peaks at 3066-3339 cm^-1^ were assigned to N-H stretching vibrations and moisture (Wang et al., 2017). The N-H absorptions of Niso overlap with the much broader O-H absorption of water molecules in MOG@IG sample due to hydrogen bonding between amino groups and water molecules. The absorption band range 712-772 cm^-1^ can be assigned to δ(O-C-O) vibrations of the ligand Niso (Zhou et al., 2016).


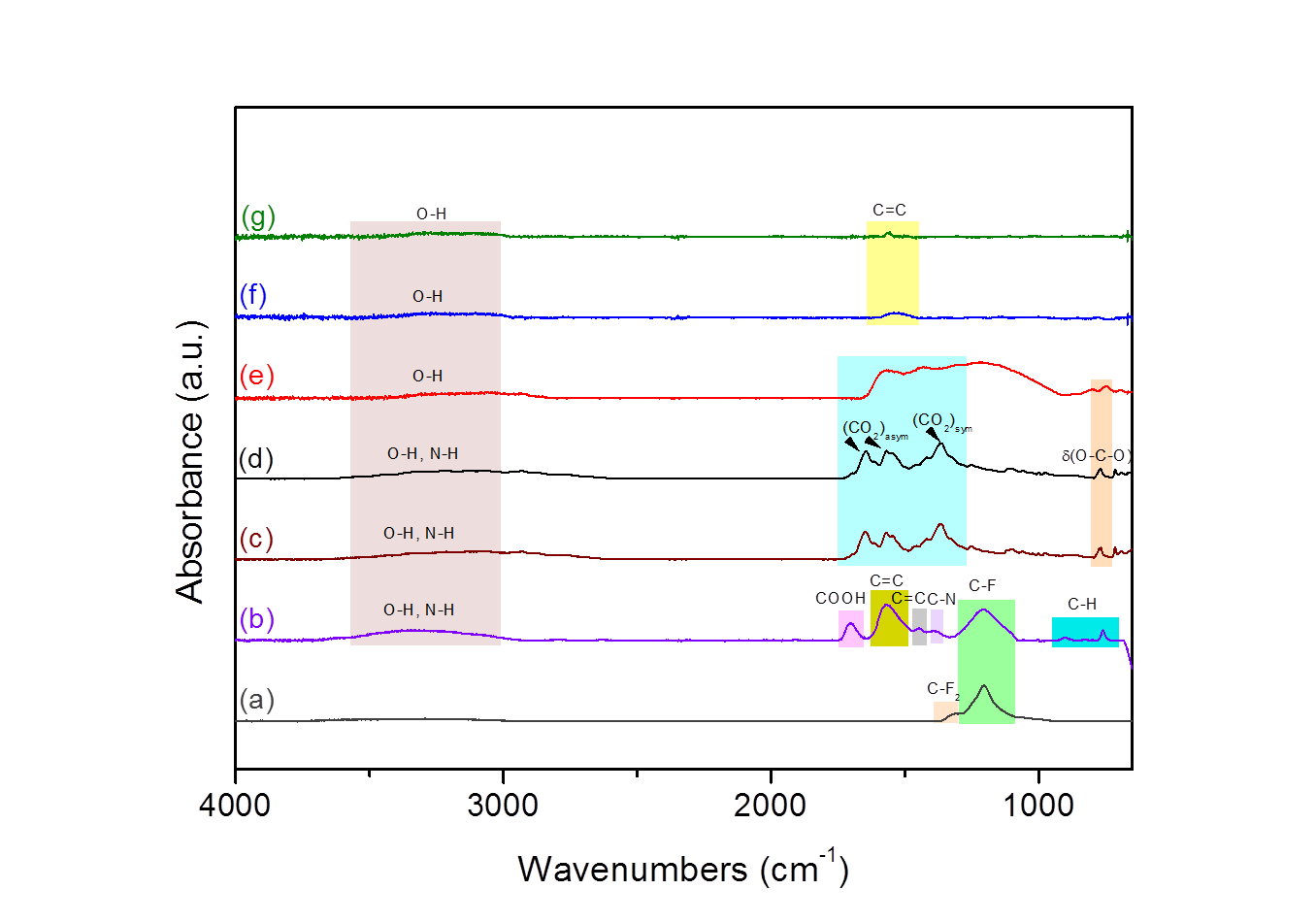


**FIGURE S1.** FTIR spectra of: (a) GrF, (b) IG, (c) MOG powder, (d) MOG@IG powder, (e) MGH-400, (f) MGH-600, and (g) MGH-800.

Wang, Y., Zhang, L. J., Zhang, R., Jin, Y., Wang, Y., Xing, et al. (2017). Porous Metal-Organic Frameworks with 5‑Aminoisophthalic Acid as Platforms for Functional Applications about High Photodegradation Efficiency of Phenol. *Cryst. Growth Des.* 17, 6531-6540. <https://doi.org/10.1021/acs.cgd.7b01190>.

Zhang, K. L., Qiao, N., Gao, H. Y., Zhou, F., and Zhang, M. (2007). Self assembly of two novel three-dimensional supramolecular networks with blue photoluminescence. *Polyhedron* 26, 2461-2469. <https://doi.org/10.1016/j.poly.2006.12.047>.

Zhou, Y. Y., Geng, B., Zhang, Z. W., and Bo, Q. B. (2016). Synthesis, structures and photoluminescence of three d^10^ 5-aminonicotinate and 5-aminoisophthalate coordination polymers with bilayer structures. *Inorg. Chim. Acta* 444, 150-158. <https://doi.org/10.1016/j.ica.2016.02.001>.

**XPS spectroscopy**

X-ray photoelectron spectroscopy (XPS) was employed in order to determine the elemental composition and chemical state of atoms in MOG@IG powder composite. The XPS survey spectra is presented in **Figure S2a** and reveals for the elements C, N, O, Fe, F the following atomic concentrations (at. %): 58.4, 7.5, 26.0, 7.0 and 1.1, respectively. The C1s XPS spectrum of MOG@IG compared to that of IG (**Figure S2b**) clearly indicates the presence of the ligand functional groups. The atomic percentage of carboxylate groups -C=O has increased by a factor of ~2.4 and C-N (or C-N/C-O) groups were doubled. The content of C-F group remains in similar level, while the sp^2^ C in C=C and sp^3^ C in C-C are reduced since the content of graphene in the composite is lower (complete details presented in **Table S1**). High resolution Fe 2p XPS spectrum (**Figure S2c**) exhibits two peaks centered at 725.4 and 711.5 eV and their satellites at 732.4 eV and 715.6 eV ascribed to Fe 2p1/2 and Fe 2p3/2 of Fe^3+^, correspondingly. These satellite peaks constitute the fingerprint of Fe^3+^ electronic structures and imply the absence of Fe^2+^. The peak separation is ~13.9 eV and can be attributed to α-Fe_2_O_3_ nodes in the MOG framework (Yu et al., 2019) equal to the peak separation for MIL-100 (Fe) (Lv et al., 2015), evidencing similar structure in accordance to XRD results. In **Figure S2d** the high resolution O 1s XPS spectrum is depicted where the peaks at 530.1, 531.8 and 533.3 eV are assigned to the Fe-O, O=C and O-C bonds in MOG@IG in accordance with literature (Yang et al., 2016).


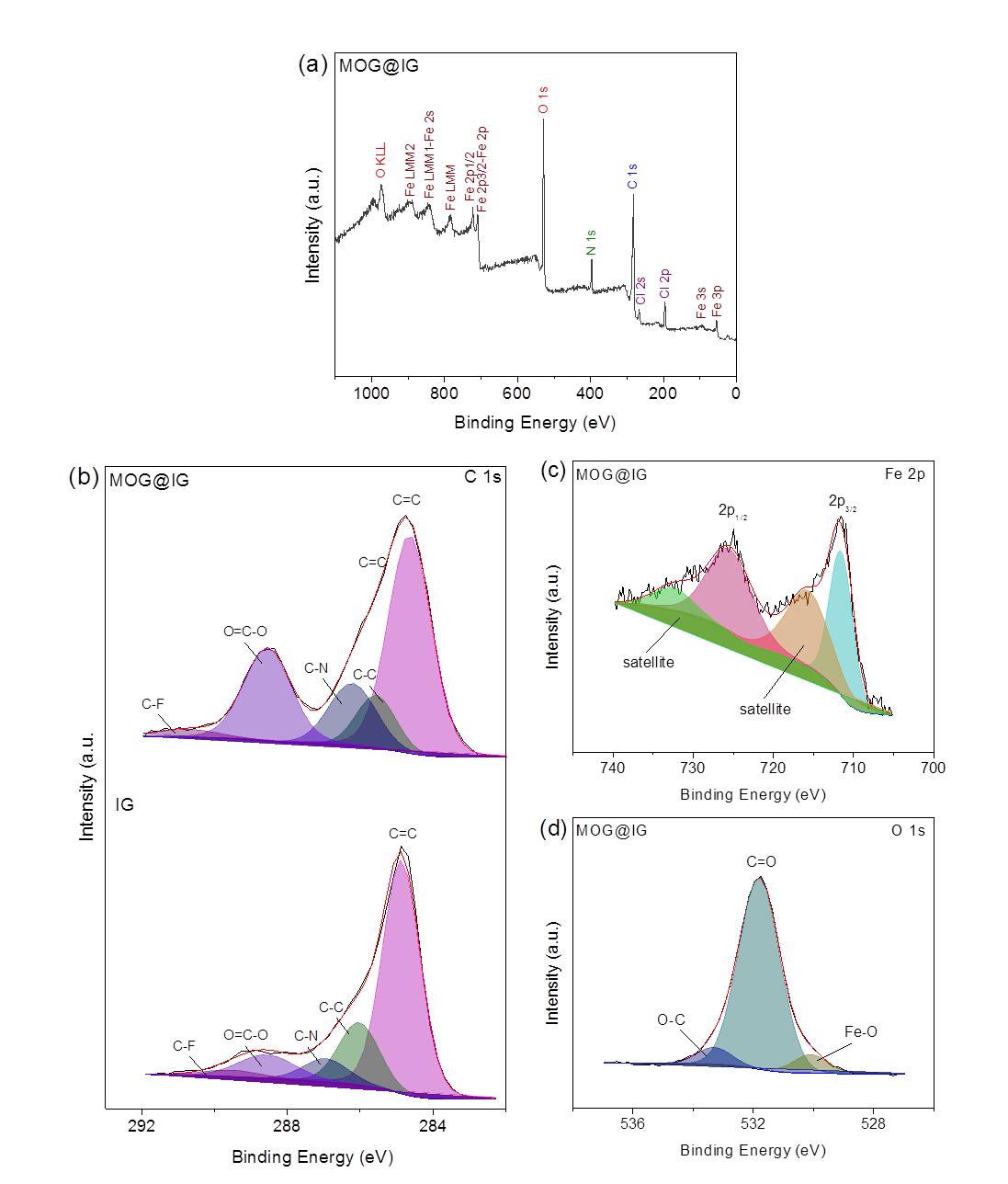


**FIGURE S2.** (a) XPS survey spectrum of MOG@IG powder; (b) High-resolution C 1s spectra of IG (bottom) and MOG@IG (top), (c) high resolution Fe 2p and (d) high resolution O1s spectra of MOG@IG.

Lv, H., Zhao, H., Cao, T., Qian, L., Wang, Y., and Zhao, G. (2015). Efficient degradation of high concentration azo-dye wastewater by heterogeneous Fenton process with iron-based metal-organic framework. *J. Molec. Catal. A Chem.* 400, 81-89. <https://doi.org/10.1016/j.molcata.2015.02.007>.

Yang, Q., Zhao, Q., Ren, S. S., Lu, Q., Guo, X., and Chen, Z. (2016). Fabrication of core-shell Fe_3_O_4_@MIL-100(Fe) magnetic microspheres for the removal of Cr(VI) in aqueous solution. *J. Solid State Chem.* 244, 25-30, <https://doi.org/10.1016/j.jssc.2016.09.010>.

Yu, D., Wu, M., Hu, Q., Wang, L., Lv, C., and Zhang, L. (2019). Iron-based metal-organic frameworks as novel platforms for catalytic ozonation of organic pollutant: Efficiency and mechanism, *J. Hazard. Mater.* 367, 456-464. <https://doi.org/10.1016/j.jhazmat.2018.12.108>.

**TABLE S1.** Atomic percentage (at. %) of various groups in IG and MOG@IG composite obtained from deconvolution of high resolution C1s XPS spectra.

| Samples | C-C (sp^2^)  ~284.7 eV | C-C (sp^3^)  ~285.8 eV | C-N  ~286.6 eV | O=C-O  ~288.5 eV | C-F  ~290.1 eV |
| --- | --- | --- | --- | --- | --- |
| IG | 62.5 | 17.8 | 7.5 | 9.6 | 2.6 |
| MOG@IG | 49.5 | 10.3 | 14.5 | 23.4 | 2.3 |

**Raman spectroscopy**

The signals at ~790 cm^-1^, ~1000 cm^-1^ and ~1595 cm^-1^ in MOG (**Figure S3, bottom**) can be attributed to the –COO- deformation mode and C-C aromatic ring vibrations, respectively. Additionally, the asymmetric and symmetric stretching vibrations of carboxylates correspond to the Raman bands at ~1416 and ~1538 cm^-1^. C-H vibration modes appear in the range 2800-3000 cm^-1^. In the Raman spectrum of MOG@IG (**Figure S3, top**) the graphene characteristics prevail as it is documented by the D and G peaks.


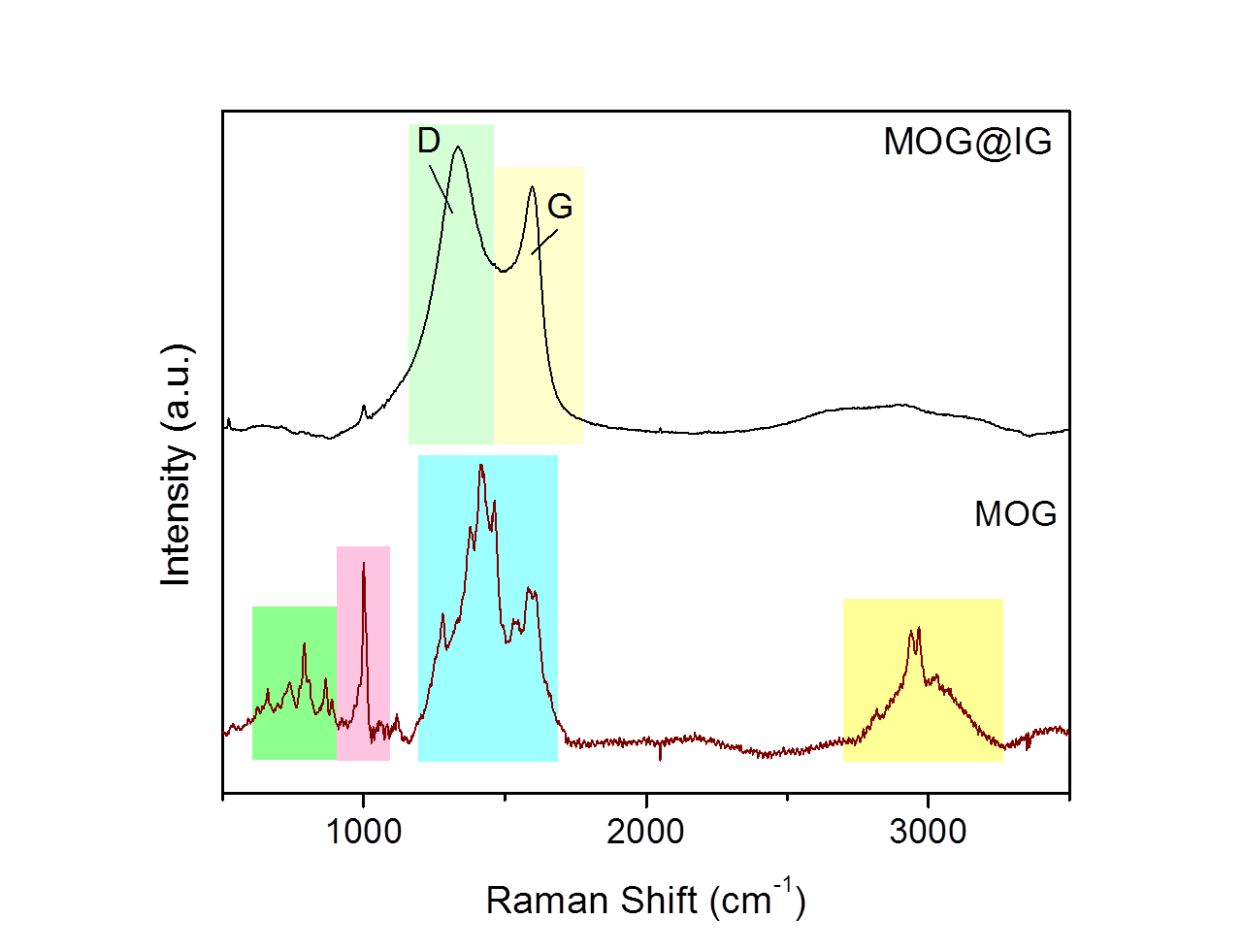


**FIGURE S3.** Raman spectra of MOG powder (bottom) and MOG@IG powder (top).


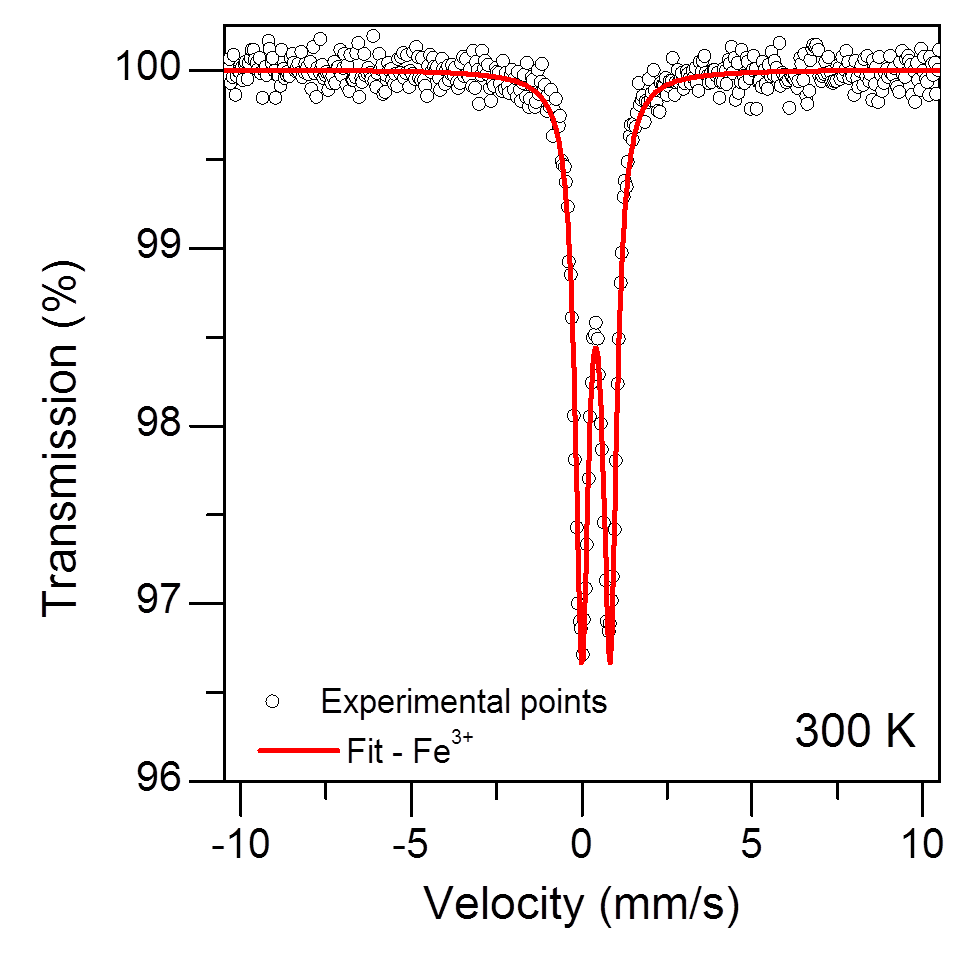


**FIGURE S4.** Mӧssbauer spectrum of MOG@IG powder.

**TABLE S2.** Values of the Mössbauer hyperfine parameters derived from the fitting procedure, where T is the temperature of the measurement, *δ* is the isomer shift, ΔE_Q_ is the quadrupole splitting, B_hf_ is the hyperfine magnetic field and RA is relative spectra area of each component. *The average quadrupole splitting derived from the distribution of the quadrupole splitting parameter (see inset in **Figure S10b**). T-sites and O-sites denote the tetrahedral and octahedral sites in the Fe_3_O_4_ crystal structure.

| **Sample** | ***T***  **(K)** | **Component** | ***δ***  **± 0.01**  **(mm/s)** | **Δ*E*_Q_**  **± 0.01**  **(mm/s)** | ***B*_hf_**  **± 0.3**  **(T)** | **RA**  **± 1**  **(%)** | **Assignment** |
| --- | --- | --- | --- | --- | --- | --- | --- |
| MOG@IG  MGH-400  MGH-600  MGH-800 | 300  300 | Doublet  Doublet  Sextet 1  Sextet 2  Sextet 3  Sextet 1  Sextet 2  Sextet 3  Sextet 4  Doublet  Sextet 1  Sextet 2 | 0.42  0.82  0.66  0.28  0.19  0.00  0.20  0.60  0.35  0.35  0.00  0.19 | 0.81  0.91*  0.00  0.00  0.00  0.00  0.00  0.00  0.00  0.89  0.00  0.00 | --  --  46.0  49.1  20.7  33.0  20.6  46.0  49.5  --  33.0  20.6 | 100  11  52  33  4  78  18  2  2  5  50  45 | Fe^3+^ ions  Fe^3+^ ions  Magnetite – O-sites  Magnetite – T-sites  Fe_3_C  nZVI  Fe_3_C  Magnetite – O-sites  Magnetite – T-sites  Fe^3+^ ions  nZVI  Fe_3_C |


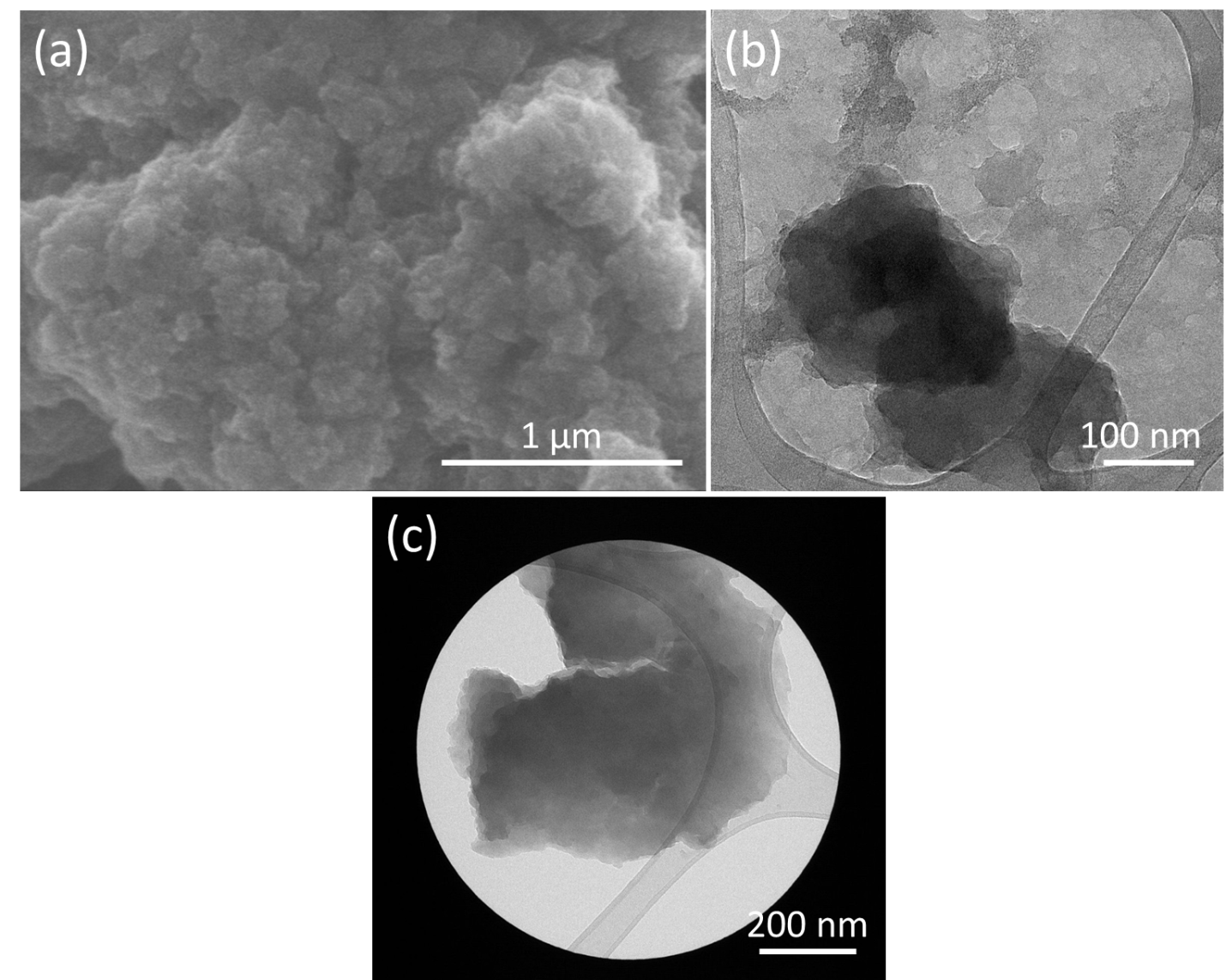


**FIGURE S5.** (a) SEM and (b) TEM micrographs of MOG@IG (before solvent removal); (c) TEM image of MOG@IG powder (after solvent removal).


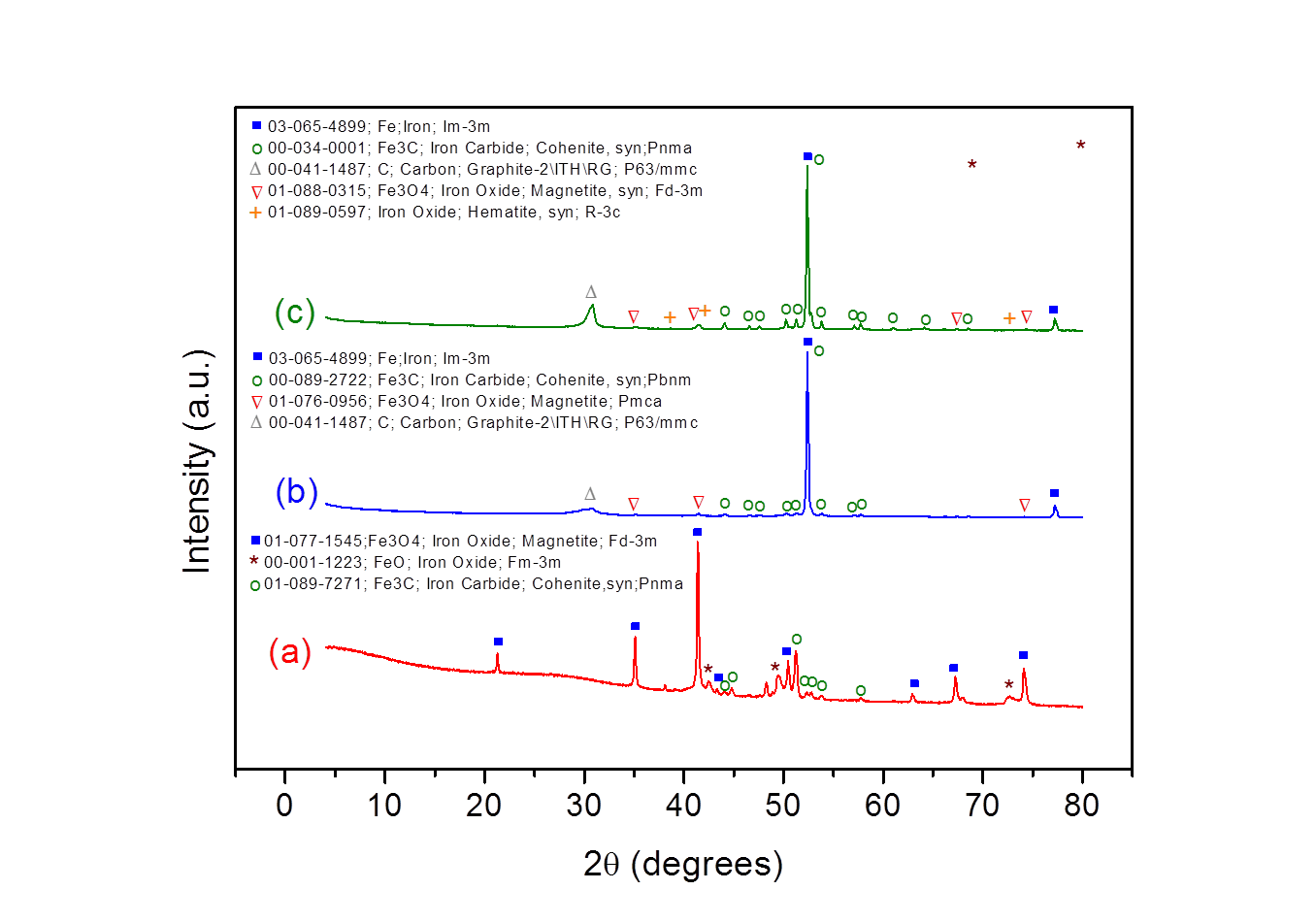


**FIGURE S6.** XRD patterns of: (a) MGH-400, (b) MGH-600 and (c) MGH-800 samples (Co-Kα radiation source, 40 kV, 30 mA, λ= 0.1789 nm).


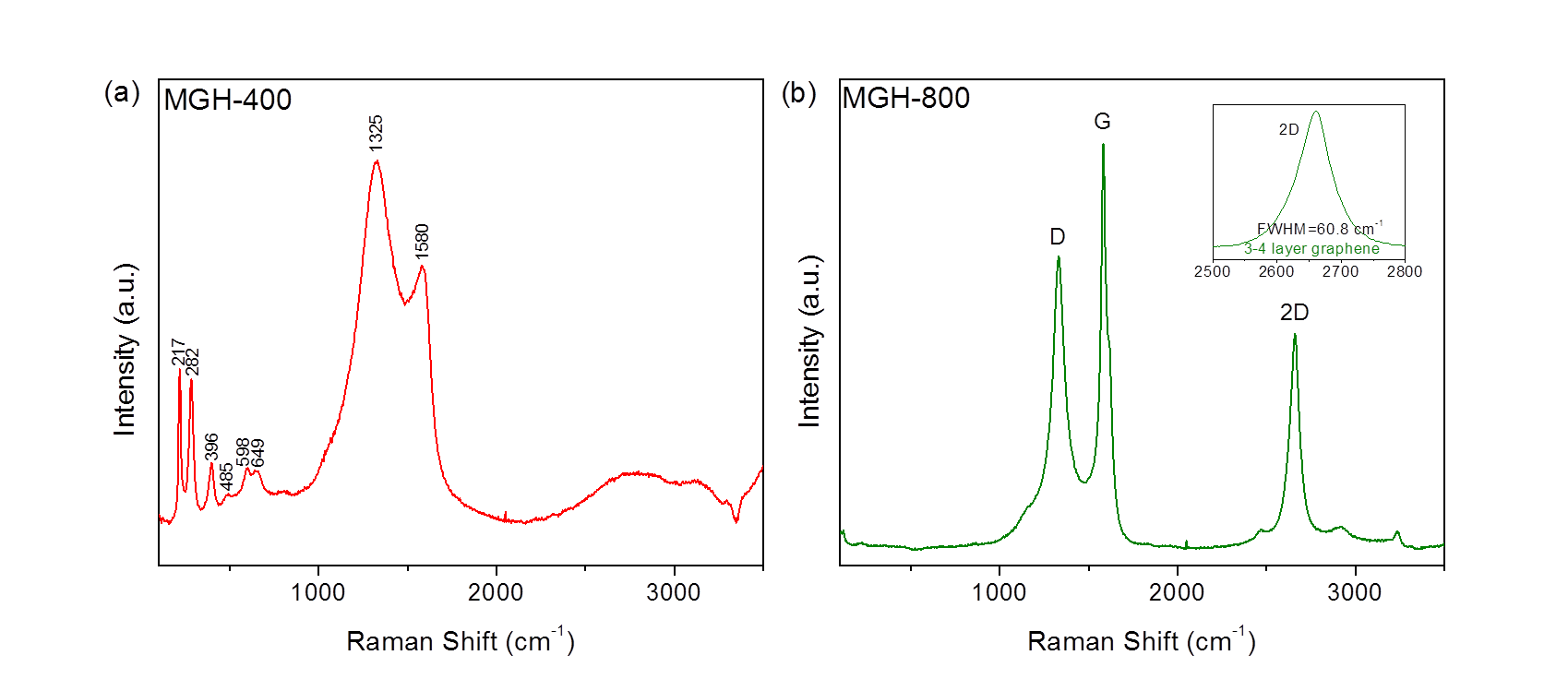


**FIGURE S7.** Raman spectra of: (a) MGH-400 and (b) MGH-800 samples (inset: 2D peak).


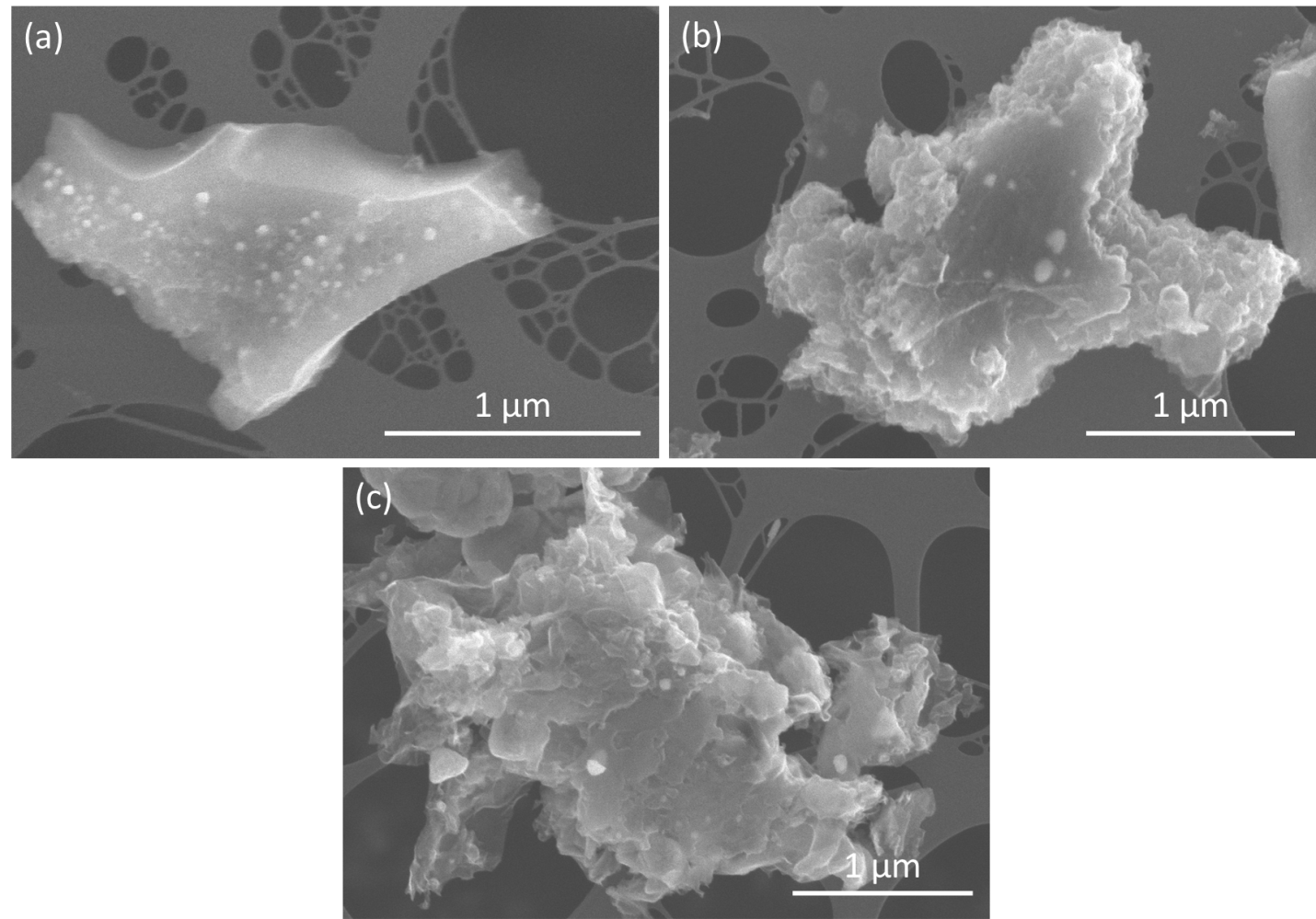


**FIGURE S8.** SEM images of: (a) MGH-400, (b) MGH-600 and (c) MGH-800 samples.


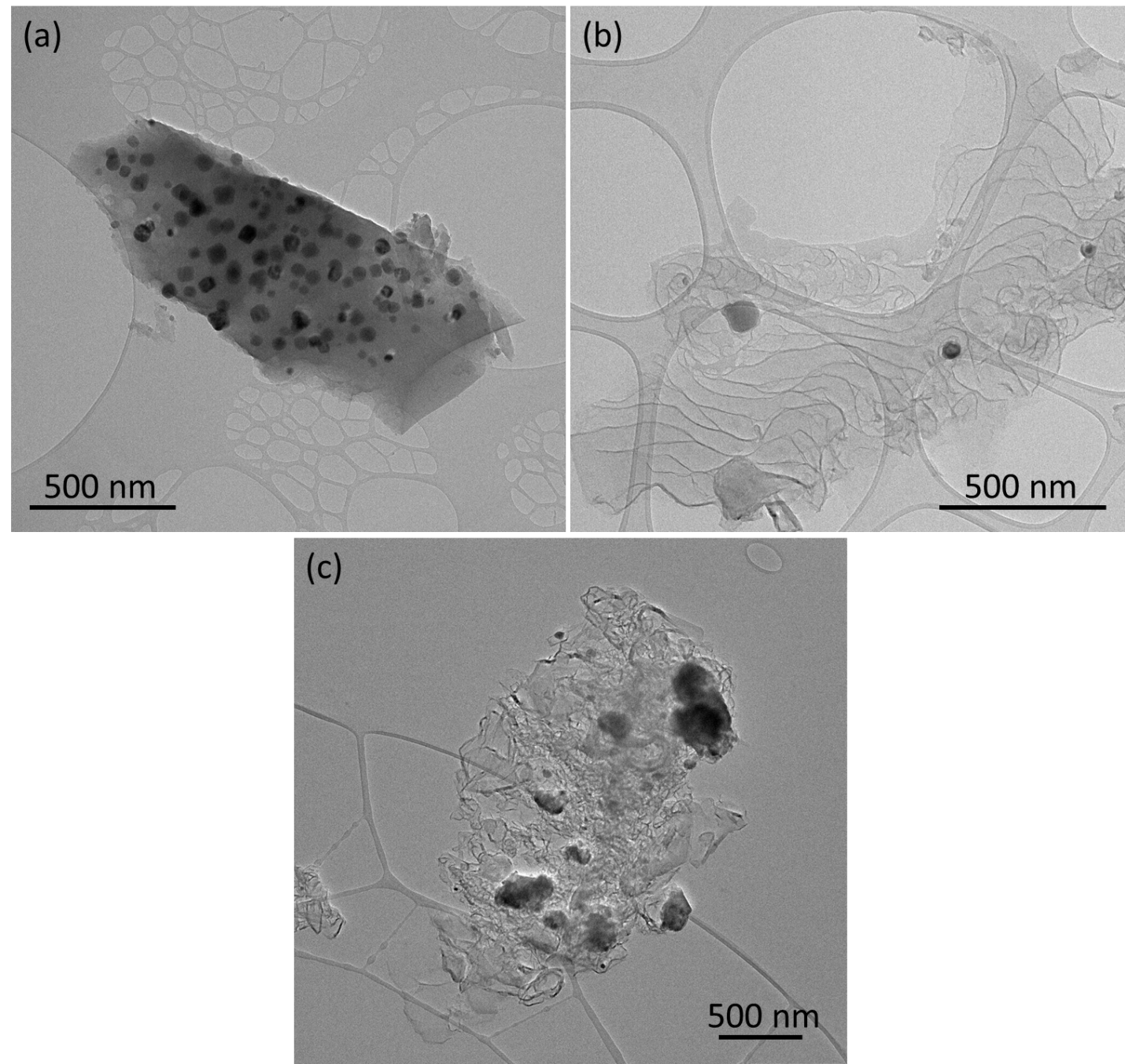


**FIGURE S9.** TEM images of: (a) MGH-400, (b) MGH-600 and (c) MGH-800 samples.


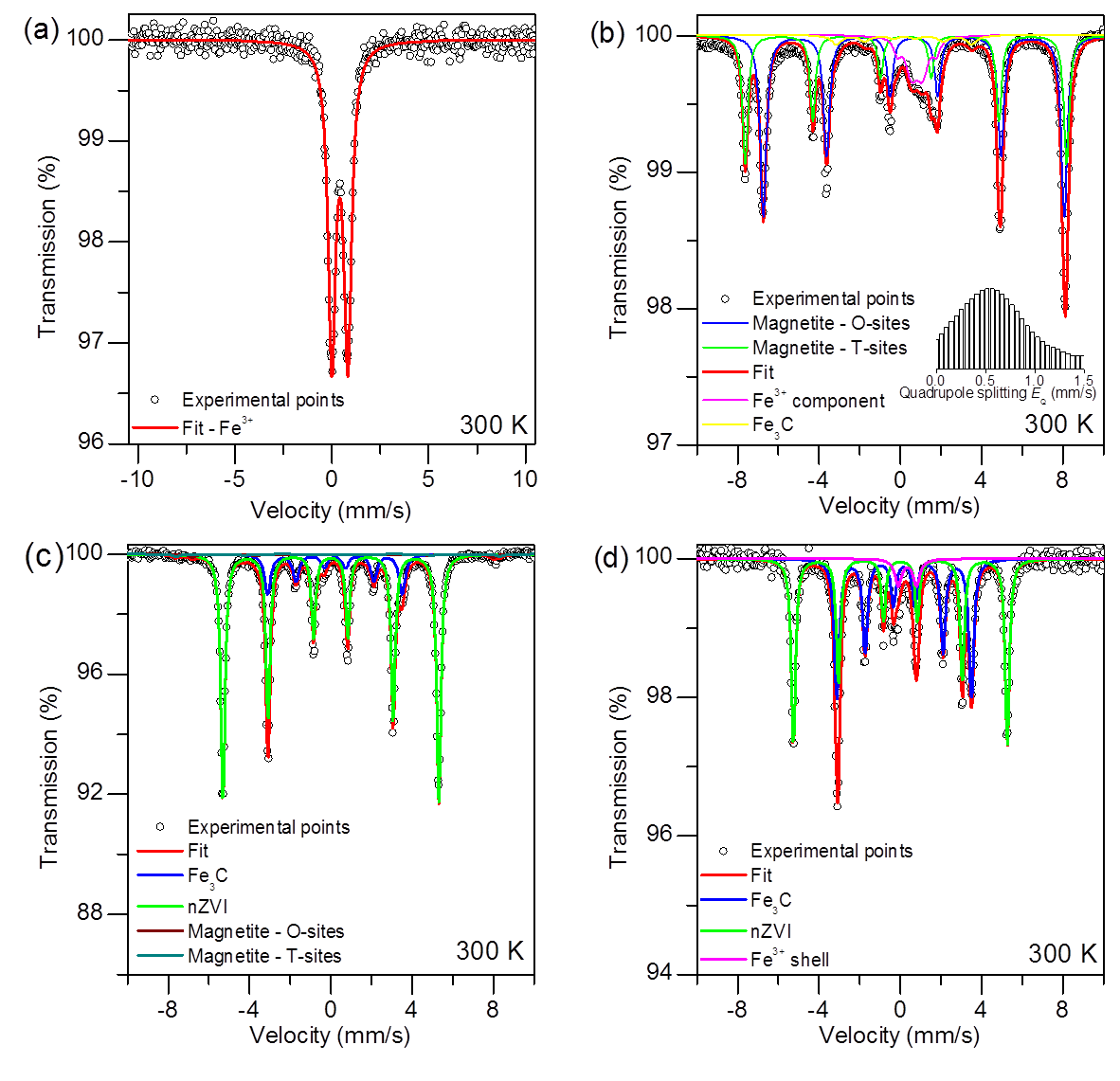


**FIGURE S10.** Mӧssbauer spectra of: (a) MOG@IG powder, (b) MGH-400, (c) MGH-600 and (d) MGH-800 samples measured at 300 K.


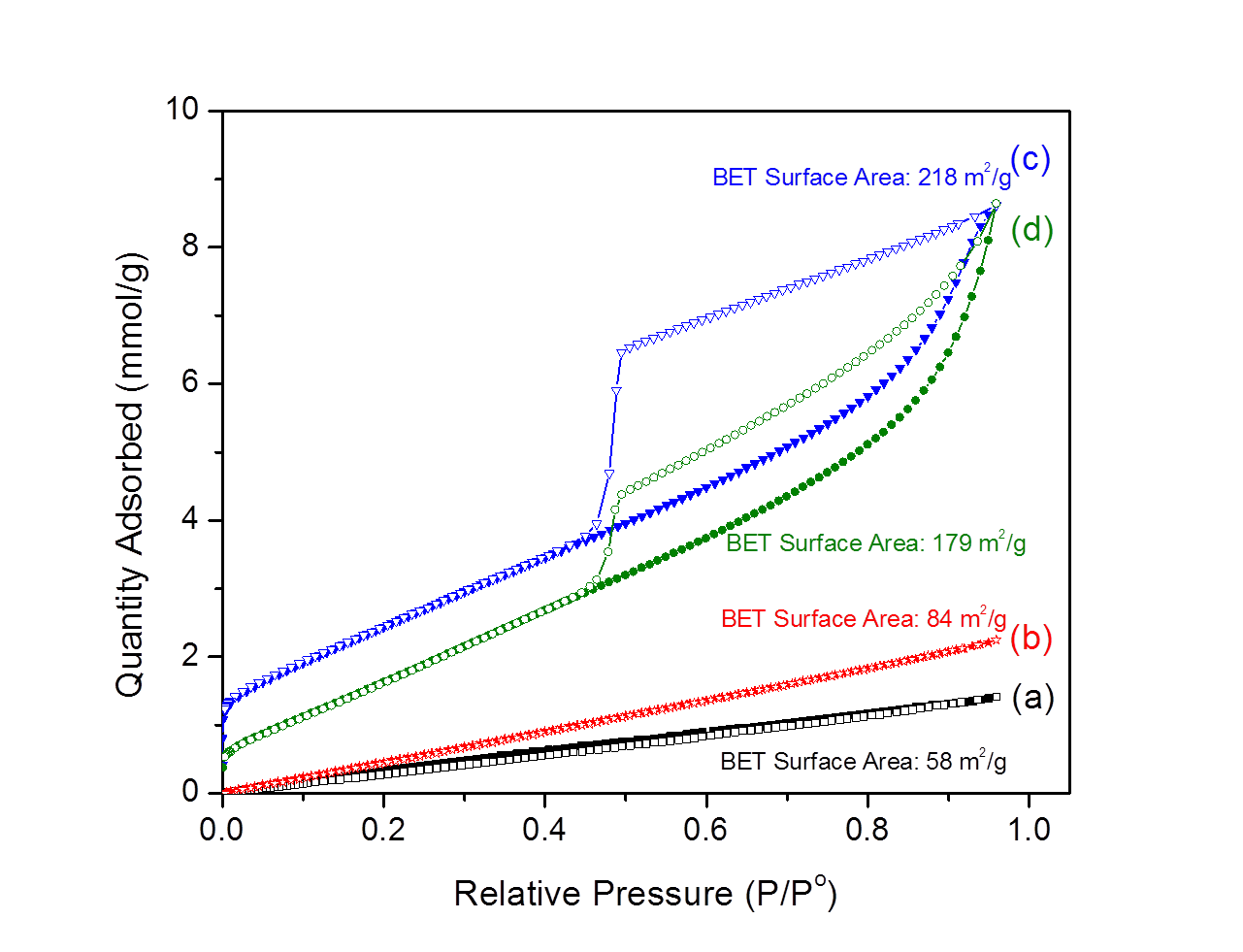


**FIGURE S11.** N_2_ adsorption/desorption isotherms obtained at -196 °C for (a) MOG@IG powder, (b) MGH-400, (c) MGH-600 and (d) MGH-800 samples and the corresponding BET Surface Areas.


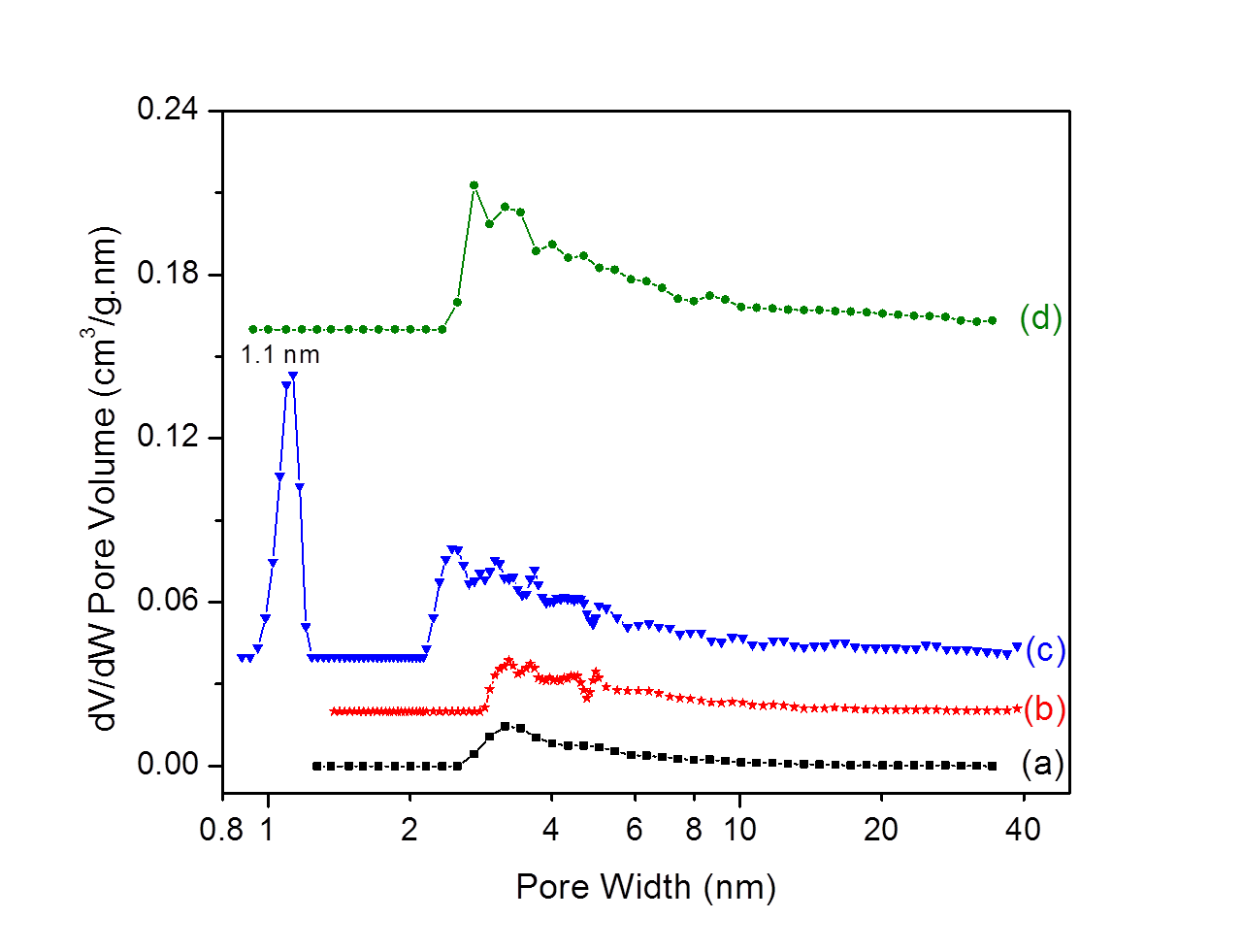


**FIGURE S12.** Pore size distributions derived using the DFT kernel for slit pores from the desorption branch of the isotherm for (a) MOG@IG powder, (b) MGH-400, (c) MGH-600 and (d) MGH-800 samples.


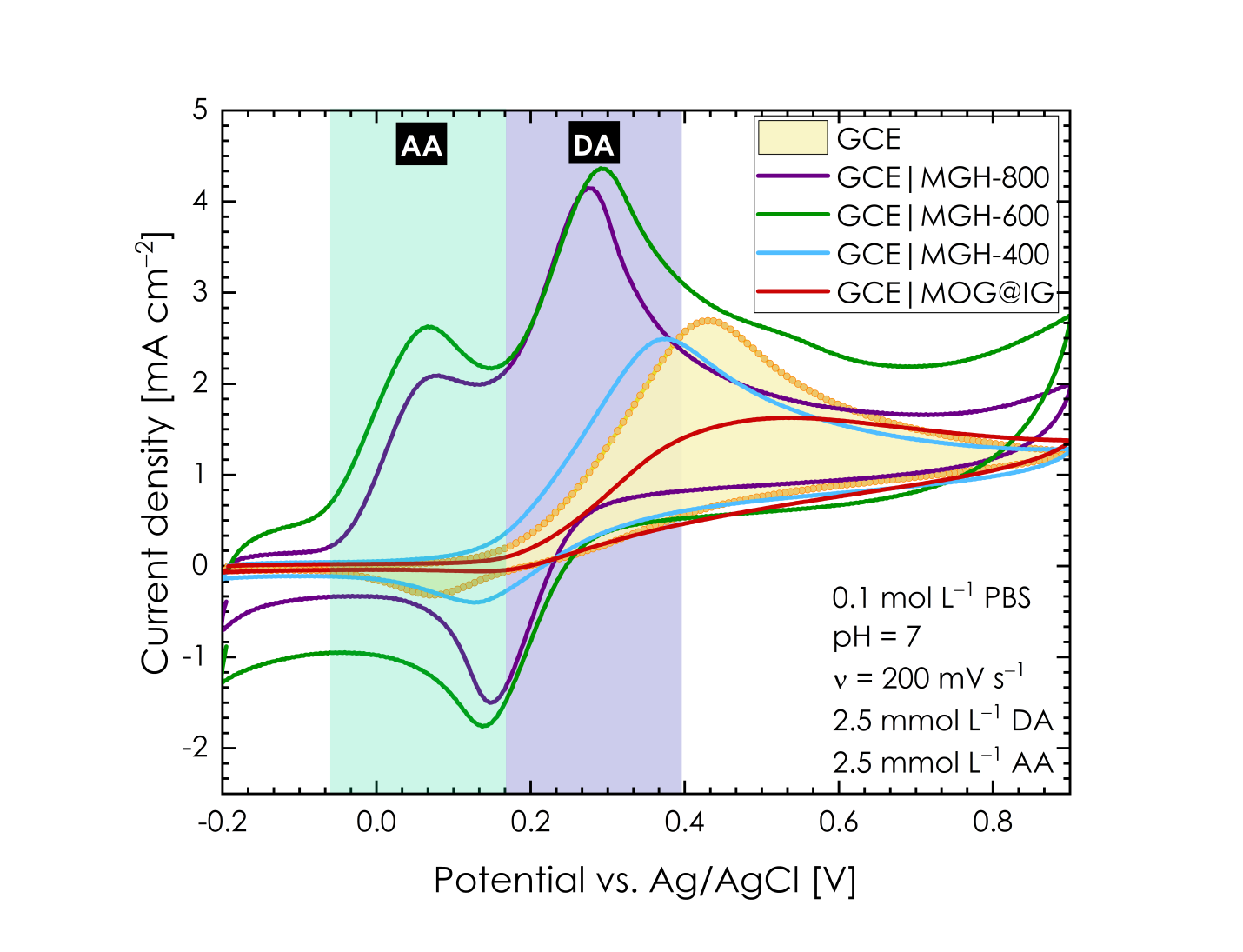


**FIGURE S13.** CV response of the bare glassy carbon electrode (GCE) and GCE modified with different samples (MOG@IG, MGH-400, MGH-600 and MGH-800) in the presence of PBS buffer (pH 7.0) containing both dopamine (c_DA_ = 2.5 mmol L^–1^) and ascorbic acid (c_AA_ = 2.5 mmol L^–1^).

**TABLE S3.** Comparison table of Raman peak intensity ratios (I_D_/I_G_), BET specific surface areas and iron derivatives present in pyrolyzed samples MGH-400, MGH-600 and MGH-800.

| **Sample** | **I_D_/I_G_** | **BET (m^2^/g)** | **Fe-derivatives** |
| --- | --- | --- | --- |
| MGH-400 | 1.4 | 84 | Fe_3_C, FeO, Fe_3_O_4_ |
| MGH-600 | 0.9 | 218 | Fe, Fe_3_C, Fe_3_O_4_ |
| MGH-800 | 0.7 | 179 | Fe, Fe_3_C, Fe_3_O_4_, Fe_2_O_3_ |


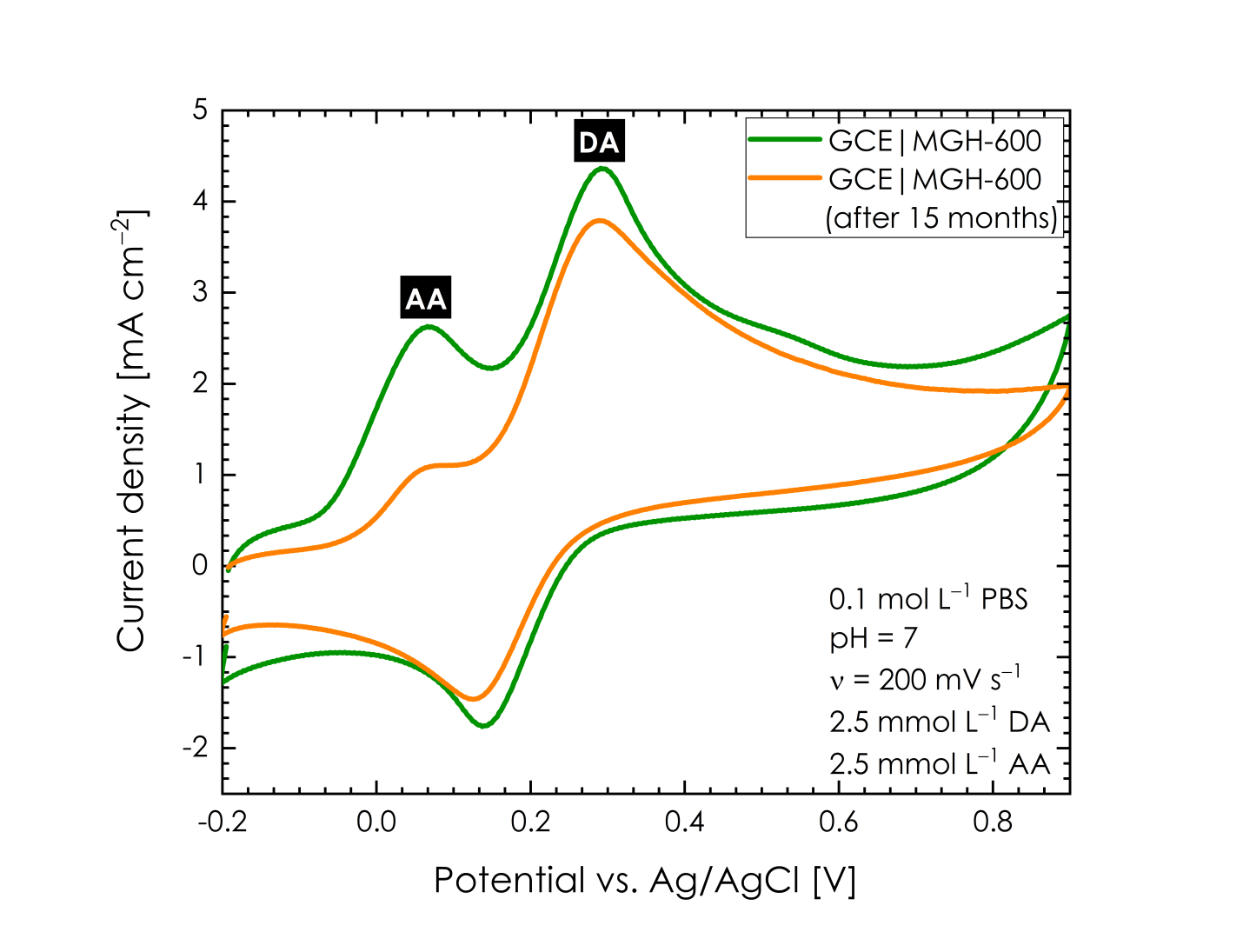


**FIGURE S14.** Stability test of MGH-600 sample. CV response of fresh MGH-600 sample (green line) and 15 months old MGH-600 sample (orange line) recorded at glassy carbon electrodes (GCEs) in the presence of PBS buffer (pH 7.0) containing both dopamine (c_DA_ = 2.5 mmol L^–1^) and ascorbic acid (c_AA_ = 2.5 mmol L^–1^).


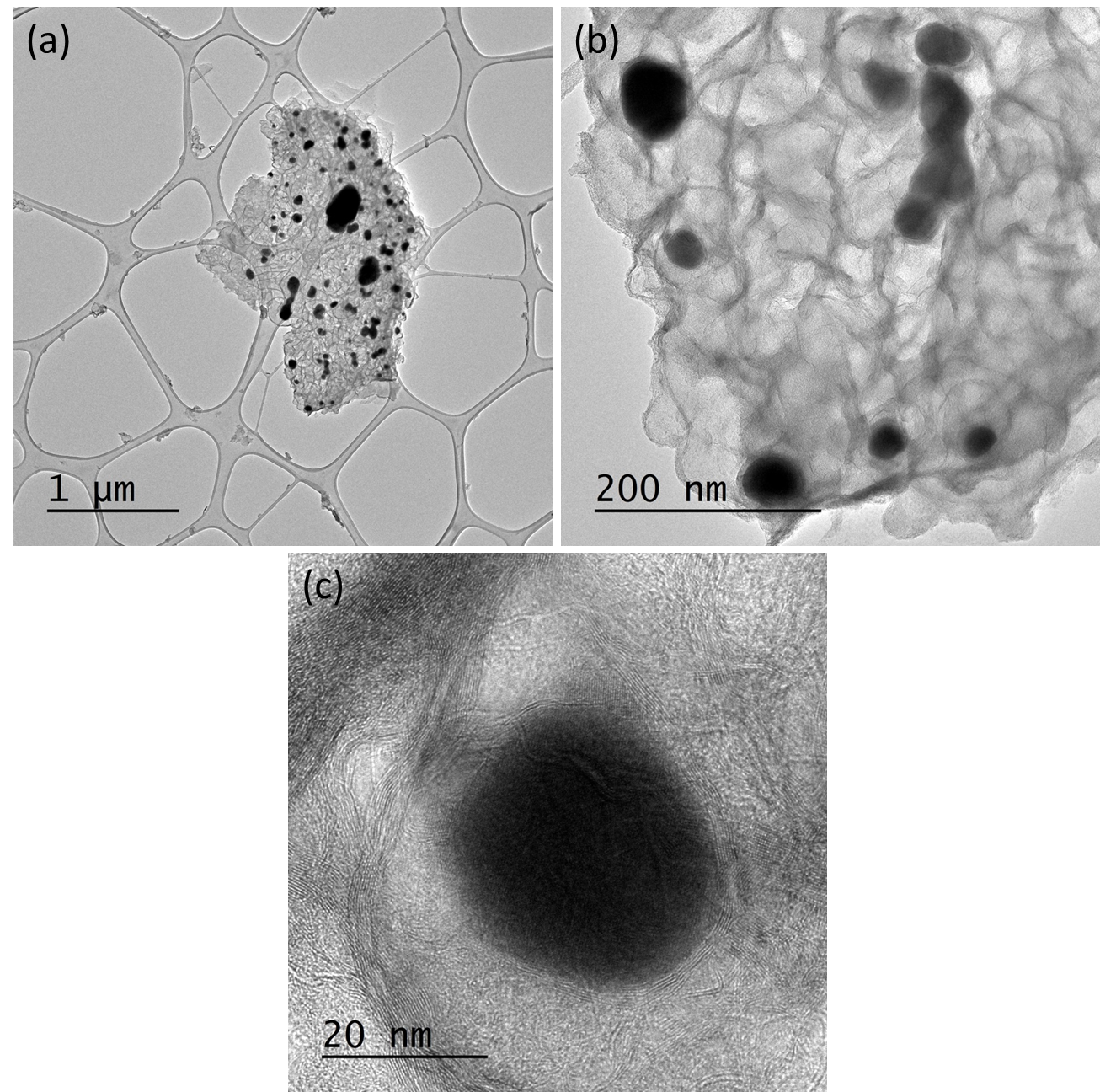


**FIGURE S15.** Microscopically investigated stability of MGH-600 sample. (a, b) TEM images of MGH-600 obtained after 15 months, (c) HRTEM image of an iron containing nanoparticle of the MGH-600 sample obtained after this term.

**TABLE S4.** List of various MOF-derived/Iron based hybrid nanocomposites (recent literature) which are compared in terms of performance in DA sensing.

| Material | Method | Linear Range (μM) | LOD (μM) | Ref. | Year |
| --- | --- | --- | --- | --- | --- |
| OFMs | Electrochemical | 0.2-115 | 0.03 | 1 | 2018 |
| Fe_3_O_4_@Cu silicate sea urchins | Electrochemical | 100-200 | 3.2 | 2 | 2018 |
| ZIF-8-AB-CS/GCE | Electrochemical | 0.1-10 | 0.004 | 3 | 2018 |
| Fe_2_O_3_ NPs/GRS | Electrochemical | 0.01-195.18 | 0.004 | 4 | 2018 |
| ACC-α-Fe_2_O_3_ | Electrochemical | 0.074-113 | 0.050 | 5 | 2018 |
| α-Fe_2_O_3_@erGO | Electrochemical | 0.25-100 | 0.024 | 6 | 2018 |
| γ-Fe_2_O_3_ nanobelts/GCE | Electrochemical | 0.6-15 | 0.6 | 7 | 2018 |
| GO-ZIF67 | Electrochemical | 0.2-80 | 0.050 | 8 | 2018 |
| Nafion/PANI/ZIF-8 | Electrochemical | 0.1-100 | 0.012 | 9 | 2018 |
| PPy/ZIF-67-MIPs/Nafion | Electrochemical | 0.08-100  100-500 | 0.0308 | 10 | 2018 |
| Eu-MOF | Fluorescent | 0-60 | 0.015 | 11 | 2019 |
| HKUST-1/GCE | Electrochemical | 12.5-175 | 0.11 | 12 | 2019 |
| Fe_3_O_4_@Au@tyrosinase | Fluorescent | 10-100 | 1 | 13 | 2019 |
| Fe_3_O_4_/GO/PG | Electrochemical | 0.30-30 | 0.18 | 14 | 2019 |
| Fe_2_O_3_/GO | Electrochemical | 0.3-30 | 0.18 | 15 | 2019 |
| sulfo-MIL-101-GPE | Electrochemical | 0.07-100 | 0.043 | 16 | 2019 |
| NH_2_-MIL-125  PA-MIL 125 | Fluorescent  Fluorescent | 0.05-0.5  0.05-0.5 | 0.01  0.005 | 17 | 2019 |
| Fe_3_O_4_-40 | Electrochemical | 0.002-0.6 | 0.008 | 18 | 2019 |
| 3D-rGO/Fe_3_O_4_/HP-β-CD/GCE | Electrochemical | 0.02-25 | 0.0067 | 19 | 2019 |
| MOF/ERGO | Electrochemical | 0.2-300 | 0.013 | 20 | 2019 |
| Au@Fe_3_O_4_/GCE | Electrochemical | 0-0.8 | 0.0027 | 21 | 2019 |
| Ni-ZIF-8/N S-CNTs/CS/GCE | Electrochemical | 8-500 | 0.93 | 22 | 2020 |
| MGH-600 | Electrochemical | 0.5-5  10-50 | 0.44 | This work | 2020 |

[1] Chen, X., Liu, Q., Liu, M., Zhang, X., Lin, S., Chen, Y., et al. (2018). Protein-templated Fe_2_O_3_ microspheres for highly sensitive amperometric detection of dopamine. *Microchim. Acta* 185, 340. <https://doi.org/10.1007/s00604-018-2876-5>.

[2] Das, A. K., Kuchi, R., Van, P. C., Sohn, Y., and Jeong, J. R. (2018). Development of an Fe_3_O_4_@Cu silicate based sensing platform for the electrochemical sensing of dopamine. *RSC Adv.* 8,31037-31047. <https://doi.org/10.1039/C8RA05885G>.

[3] Jin, Y. F., Ge,C. Y., Li, X. B., Zhang, M., Xu, G. R., and Li, D. H. (2018). A sensitive electrochemical sensor based on ZIF-8–acetylene black–chitosan nanocomposites for rutin detection. *RSC Adv.* 8, 32740-32746. <https://doi.org/10.1039/C8RA06452K>.

[4] Kokulnathan, T., Anthuvan, A. J., Chen, S. M., Chinnuswamy, V., and Kadirvelu, K. (2018). Trace level electrochemical determination of the neurotransmitter dopamine in biological samples based on iron oxide nanoparticle decorated graphene sheets. *Inorg. Chem. Front.* 5, 705-718. <https://doi.org/10.1039/C7QI00716G>.

[5] Mahesh, K. P. O., Shown, I., Chen, L. C., Chen, K. H., and Tai, Y. (2018). Flexible sensor for dopamine detection fabricated by the direct growth of α-Fe_2_O_3_ nanoparticles on carbon cloth. *Appl. Surf. Sci.* 427, 387-395. <https://doi.org/10.1016/j.apsusc.2017.08.168>.

[6] Mathew, G., Dey, P., Das, R., Chowdhury, S. D., Das, M. P., Veluswamy, P., et al. (2018). Direct electrochemical reduction of hematite decorated graphene oxide (α- Fe_2_O_3_@erGO) nanocomposite for selective detection of Parkinson's disease biomarker, *Biosens. Bioelectron.* 115, 53-60. <https://doi.org/10.1016/j.bios.2018.05.024>.

[7] Sundar, S., Venkatachalam, G., and Kwon, S. J. (2018). Sol-Gel Mediated Greener Synthesis of γ-Fe_2_O_3_ Nanostructures for the Selective and Sensitive Determination of Uric Acid and Dopamine. *Catalysts* 8, 512; doi:10.3390/catal8110512.

[8] Tang, J., Jiang, S., Liu, Y., Zheng, S., Bai, L., Guo, J., et al. (2018). Electrochemical determination of dopamine and uric acid using a glassy carbon electrode modified with a composite consisting of a Co(II)-based metalorganic framework (ZIF-67) and graphene oxide. *Microchim. Acta* 185, 486. <https://doi.org/10.1007/s00604-018-3025-x>

[9] Yuan, Y., Xia, J., Zhang, F., Wang, Z., and Liu, Q. (2018). Nafion/polyaniline/Zeolitic Imidazolate Framework-8 nanocomposite sensor for the electrochemical determination of dopamine. *J. Electroanal. Chem.* 824, 147-152. <https://doi.org/10.1016/j.jelechem.2018.07.048>.

[10] Zhang, W., Duan, D., Liu, S., Zhang, Y., Leng, L., Li, X., et al. (2018). Metal-organic framework-based molecularly imprinted polymer as a high sensitive and selective hybrid for the determination of dopamine in injections and human serum samples. *Biosens. Bioelectron.* 118, 129-136. <https://doi.org/10.1016/j.bios.2018.07.047>.

[11] Du, Q., Wu, P., Dramou, P., Chen, R., and He, H. (2019). One-step fabrication of a boric acid-functionalized lanthanide metal–organic framework as a ratiometric fluorescence sensor for the selective recognition of dopamine. *New J. Chem.* 43, 1291-1298. <https://doi.org/10.1039/C8NJ05318A>.

[12] Sofi, F. A., Bhat, M. A., and Majid, K. (2019). Cu^2+^-BTC based metal–organic framework: a redox accessible and redox stable MOF for selective and sensitive electrochemical sensing of acetaminophen and dopamine. *New J. Chem.* 43, 3119-3127. <https://doi.org/10.1039/C8NJ06224B>.

[13] Arkan, E., Karami, C., and Rafipur, R. (2019). Immobilization of tyrosinase on Fe_3_O_4_@Au core-shell nanoparticles as bio‑probe for detection of dopamine, phenol and catechol. *J. Biol. Inorg. Chem.* 24, 961-969. <https://doi.org/10.1007/s00775-019-01691-0>.

[14] Cai, L., Hou, B., Shang, Y., Xu, L., Zhou, B., Jiang, X., et al. (2019). Synthesis of Fe_3_O_4_/graphene oxide/pristine graphene ternary composite and fabrication electrochemical sensor to detect dopamine and hydrogen peroxide. *Chem. Phys. Lett.* 736, 736, 136797. <https://doi.org/10.1016/j.cplett.2019.136797>.

[15] Cai, Z., Ye, Y., Wan, X., Liu, J., Yang, S., Xia, Y., et al. Morphology–Dependent Electrochemical Sensing Properties of Iron Oxide–Graphene Oxide Nanohybrids for Dopamine and Uric Acid. *Nanomaterials* 9, 835. <https://doi.org/10.3390/nano9060835>.

[16] Gao, L. L., Sun, W. J., Yin, X. M., Bu, R., and Gao,W. Q. (2019). Graphite paste electrodes modified with a sulfo-functionalized metal-organic framework (type MIL-101) for voltammetric sensing of dopamine, *Microchim. Acta* 186, 762. <https://doi.org/10.1007/s00604-019-3943-2>.

[17] George, P., and Chowdhury, P. (2019). NH_2_-MIL-125(Ti) and its emeraldine functionalized derivative as a chemical sensor for effective detection of dopamine. *Micropor. Mesopor. Mater.* 288, 109591. <https://doi.org/10.1016/j.micromeso.2019.109591>.

[18] Huang, Y., Zhang, Y., Liu, D., Li, M., Yu, Y., Yang, W., et al. (2019). Facile synthesis of highly ordered mesoporous Fe_3_O_4_ with ultrasensitive detection of dopamine. *Talanta* 201, 511-518. <https://doi.org/10.1016/j.talanta.2019.01.099>.

[19] Liang, W., Rong, Y., Fan, L., Zhang, C., Dong, W., Li, J., et al. (2019). Simultaneous electrochemical sensing of serotonin, dopamine and ascorbic acid by using a nanocomposite prepared from reduced graphene oxide, Fe_3_O_4_ and hydroxypropyl-β-cyclodextrin. *Microchim. Acta* 186, 751. <https://doi.org/10.1007/s00604-019-3861-3>.

[20] Ma, B., Guo, H., Wang, M., Li, L., Jia, X., Chen, H., et al. (2019). Electrocatalysis of Cu-MOF/Graphene Composite and its Sensing Application for Electrochemical Simultaneous Determination of Dopamine and Paracetamol. *Electroanalysis* 31, 1002-1008. <https://doi.org/10.1002/elan.201800890>.

[21] Thamilselvan, A., Manivel, P., Rajagopal, V., Nesakumar, N., and Suryanarayanan, V. (2019). Improved electrocatalytic activity of Au@Fe_3_O_4_ magnetic nanoparticles for sensitive dopamine detection, *Colloids Surf. B* 180, 1-8. <https://doi.org/10.1016/j.colsurfb.2019.04.034>.

[22] Yao, W., Guo, H., Liu, H., Li, Q., Wu, N., Li, L., et al. (2020). Highly electrochemical performance of Ni-ZIF-8/ N S-CNTs/CS composite for simultaneous determination of dopamine, uric acid and L-tryptophan. *Microchem. J.* 152, 104357. <https://doi.org/10.1016/j.microc.2019.104357>.

**ABBREVIATIONS**

*OFMs:* Olive-like Fe_2_O_3_ microspheres.

*Fe_3_O_4_@Cu silicate sea urchins:* sea urchin like Fe3O4@Cu silicate core–shell nanocomposites.

*ZIF-8-AB-CS/GCE:* Glassy carbon electrode (GCE) modified with zeolitic imidazolate framework-8 (ZIF-8) and acetylene black (AB) in the presence of chitosan (CS).

*Fe_2_O_3_ NPs/GRS:* Iron oxide nanoparticle-(Fe_2_O_3_ NP) capped graphene sheet (GRS) modified glassy carbon electrode (GCE).

*ACC-α-Fe_2_O_3_:* Porous α-Fe_2_O_3_ nanoparticles directly grown on acid treated carbon cloth (ACC) using a hydrothermal method.

*α-Fe_2_O_3_@erGO:* Magnetic hematite (α-Fe_2_O_3_) decorated electrochemically reduced graphene oxide (α-Fe_2_O_3_@erGO) nanocomposite.

*γ-Fe_2_O_3_ nanobelts/GCE:* γ-Fe_2_O_3_ nanostructures modified glassy carbon electrode (GCE).

*GO-ZIF67:* Composite consisting of a Co(II)-based metalorganic framework (ZIF-67) and graphene oxide.

*Nafion/PANI/ZIF-8:* Nafion/polyaniline/Zeolitic Imidazolate Framework-8 nanocomposite fabricated on the surface of glassy carbon electrode (GCE).

*PPy/ZIF-67-MIPs/Nafion/GCE:* Molecular imprinting polymer (MIP) sensor fabricated based on polypyrrole (PPy)/ZIF-67/Nafion hybrid modified glassy carbon electrode (GCE).

*Eu-MOF:* Boric acid-modified lanthanide metal–organic framework.

*HKUST-1/GCE:* HKUST-1 modified glassy carbon electrode (GCE).

*Fe_3_O_4_@Au@tyrosinase:* Bio-probe based on the immobilized tyrosinase on Fe_3_O_4_@Au core-shell nanoparticles.

*Fe_3_O_4_/GO/PG:* Fe_3_O_4_/graphene oxide (GO)/pristine graphene (PG) ternary composite.

*Fe_2_O_3_/GO:* Discal Fe_2_O_3_ NPs coupled with graphene oxide (GO) nanosheets casted on glassy carbon electrode (GCE).

*sulfo-MIL-101-GPE:* Sulfo-functionalized metal-organic framework (type MIL-101) modified graphite paste electrode (GPE).

*NH_2_-MIL-125:* NH_2_ functionalized MIL-125 (Ti) Metal Organic Framework (MOF).

*PA-MIL 125:* Emeraldine–NH_2_–MIL-125(Ti).

*Fe_3_O_4_-40:* Ordered mesoporous Fe_3_O_4_ with high surface area modified glassy carbon electrode.

*3D-rGO/Fe_3_O_4_/HP-β-CD/GCE:* Nanocomposite prepared from reduced graphene oxide, Fe_3_O_4_ and hydroxypropyl-β-cyclodextrin deposited on glassy carbon electrode (GCE).

*MOF/ERGO:* A nanocomposite of HKUST-1 (MOF) and electroreduction graphene oxide (ERGO) deposited on glassy carbon electrode (GCE).

*Au@Fe_3_O_4_/GCE:* Gold (Au) nanoparticles (NPs) systematically decorated with magnetic Fe_3_O_4_ nanocomposites deposited on glassy carbon electrode (GCE).

*Ni-ZIF-8/N S-CNTs/CS/GCE:* Ni-ZIF-8, N S-CNTs (carbon nanotubes), chitosan composite deposited on glassy carbon electrode (GCE).
